# Intervention on modifiable lifestyle and physiological factors via variational autoencoder reveals changes in functional connectivity-mediated risk for Alzheimer’s disease

**DOI:** 10.1162/IMAG.a.1408

**Published:** 2026-10-22

**Authors:** Anton Orlichenko, Shengxian Ding, Emily Johns, Zhiling Gu, Xinyuan Tian, Xiaoxuan Li, Yize Zhao

**Affiliations:** Department of Biostatistics, Yale School of Public Health, New Haven, CT, United States; Department of Biomedical Engineering, Tulane University, New Orleans, LA, United States

**Keywords:** Alzheimer’s disease, data integration, functional connectivity, lifespan, modifiable risk factors, variational autoencoder

## Abstract

Alzheimer’s disease (AD) remains without effective treatment, largely due to the fact that clinical symptoms emerge only after decades of silent pathological progression. It is urgently needed to identify modifiable risk factors in earlier life stages, when preventive interventions may still be effective. Functional connectivity (FC) has emerged as a promising neuromarker for both neurodegenerative processes and behavioral traits, making it a potential bridge between early-life health profiles and late-life AD risk. In this work, we introduce a novel integrative framework, called InterventionVAE, that models how early-life lifestyle and physiological factors influence AD risk through their impact on brain FC. Our approach combines a modified variational autoencoder (VAE) that simulates FC changes under interventions with a predictive model that estimates AD risk based on FC patterns. This design enables training of the generative and predictive components under different datasets and populations, with FC acting as the bridge between early-life modifiable factors and late-life disease risk. Applying InterventionVAE to data from the Human Connectome Project (HCP), UK Biobank (UKB), and Alzheimer’s Disease Neuroimaging Initiative (ADNI), we validate its ability to capture known risk factors, such as age and polygenic risk score, on FC-mediated AD risk. We also identify earlier-life modifiable factors including tobacco use, sleep quality, physical activity, and weight/BMI that significantly influence AD risk. Notably, we observe a U-shaped relationship between blood pressure and AD risk, and highlight the brain visual and somatomotor networks as key mediators of risk through FC. Our approach provides a powerful tool for investigating the effect pathways linking early-life interventions to neurodegenerative outcomes, with broad applicability to other brain-related disorders.

## 1 Introduction

Alzheimer’s disease (AD) is a progressive neurodegenerative disease characterized by cognitive decline, loss of memory, and impaired reasoning skills (Safiri et al., 2024). The pathological hallmarks of AD are widely recognized to include the accumulation of amyloid-β and tau peptide (Y. Chen & Yu, 2023; M. P. Murphy & LeVine, 2010). An estimated 6.5 million Americans are currently living with AD, imposing substantial economic burdens that amount to hundreds of billions of dollars annually. Existing treatments, such as acetylcholinesterase inhibitors, non-steroidal anti-inflammatory compounds, sex steroid hormones, and antioxidants (Allain et al., 2007), demonstrate limited efficacy in altering the progression of AD and mild cognitive impairment (MCI). Moreover, AD symptoms typically manifest many years after the initial onset of pathological changes, meaning that therapeutic interventions often occur too late to be fully effective (Thangwaritorn et al., 2024). Consequently, there has been significant research interest in developing effective interventions during early-life stages, before clinical symptoms manifest (Gandy et al., 2017; Seifan et al., 2015). Early interventions well before the onset of noticeable cognitive symptoms can be essential for altering disease outcomes timely.

The stages of AD progression include the preclinical, prodromal, and patent dementia periods (Vermunt et al., 2019). The preclinical AD stage can begin as early as midlife and span decades without overt symptoms, despite accumulating pathological features such as amyloid plaques and neurofibrillary tangles in the brain (Thal et al., 2013). The prodromal stage involves MCI, where measurable deficits are present but daily independence is maintained (Albert et al., 2011), followed by dementia, characterized by severe impairments in cognition and everyday functioning. The long, symptom-free preclinical stage (averaging 10 years) poses a major challenge to early detection. Most studies attempting to capture this stage must enroll large cohorts of cognitively normal individuals, of whom only about 10% will eventually develop AD (Bennett et al., 2018; Dhana et al., 2023; Petersen et al., 2010). This makes such research inefficient and resource-intensive. As a result, there is growing interest in using biomarkers to more precisely identify individuals at risk during these early stages (Millar et al., 2022; Risacher & Saykin, 2013). Note that the term AD progression should be reserved for findings supported by longitudinal or pathological evidence, rather than implying that our FC model directly measures progression. This distinction is consistent with the manuscript’s explicit acknowledgment that FC is an intermediate representation linking early-life factors to later AD risk and that the direct effect on AD outcome is unobserved, as seen from our assumptions made in Figure 1.

**Fig. 1.**
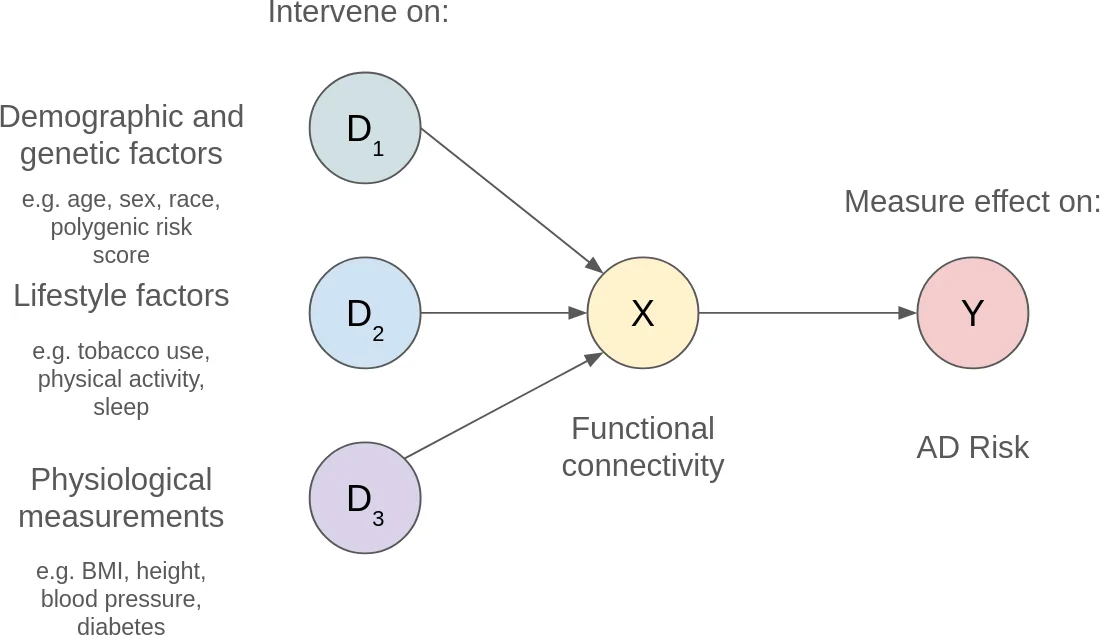
High-level overview of the causal effects in our model. We assume that demographic, genetic, and lifestyle factors D all have an effect on FC X. Additionally, FC is assumed to be representative of cognitive changes occurring in AD represented by risk Y. By creating a model that is able to synthesize FC dependent on D, we can quantify the increased or decreased risk of AD based on disentangled interventions on D. The direct effect of D on Y is unobserved. Note that these assumptions always modify the conclusions that can be gleaned from the effect of lifestyle factors on AD since a full causal analysis including FC profiles is untractable with presently available datasets.

Among these biomarkers, neuroimaging, including functional MRI (fMRI), has shown great promise to reveal altered activation patterns in memory-related brain regions among individuals at risk (Noh et al., 2023). Functional connectivity (FC), an fMRI-based imaging feature derived to characterize network-level temporal synchronization of activity, has emerged as a particularly powerful neuromarker. FC consistently reveals network-level disruptions during the transition from cognitively normal aging to MCI (Penalba-Sánchez et al., 2022), and has demonstrated strong predictive accuracy in identifying individuals likely to progress from prodromal AD to dementia (Noh et al., 2023). Beyond AD, FC serves as a robust individual fingerprint, capturing both normative traits such as age (Hu et al., 2019), sex (İçer et al., 2020), race (Orlichenko et al., 2023), and cognitive ability (Qu et al., 2024), as well as deviations seen in disorders like ADHD (Castellanos & Aoki, 2016), schizophrenia (Collin et al., 2020), and ASD (Sung & Park, 2024). Given the sensitivity of FC to both normal variation and disease states, and its quantifiable progression across AD stages, we propose using FC as a bridge to link early-life behaviors and physiological traits to later AD risk. Critically, most current AD studies focus on older adults who are already on the disease trajectory (Bennett et al., 2018; Petersen et al., 2010), limiting our understanding of how modifiable early-life factors shape long-term vulnerability.

In this study, we leverage FC as an intermediate neurobiological representation to bridge the potential causal pathway from early-life behaviors and physiological measurements to late-life AD risk. By rigorously modeling how modifiable factors shape FC and how FC influences AD vulnerability across studies over lifespan, we aim to elucidate covariates by which early interventions may alter long-term disease outcomes. As illustrated in Figure 1, we develop a behavior- and lifestyle factor-conditioned variational autoencoder (VAE) to model how modifiable factors influence FC and, in turn, AD risk. In the current literature, this kind of analytical objective has been mainly achieved by Mendelian randomization (MR) as discussed in Section 2, which tends to be constrained by the availability of strong genetic instrumental variables for diverse behaviors or lifestyle factors (Korologou-Linden et al., 2022; Sanderson et al., 2022), known as the weak IV problem (Andrews et al., 2019). This limitation has hindered efforts to establish causal links between early-life exposures and late-life clinical outcomes in general. In contrast, behaviors and lifestyle factors are strongly correlated with FC endophenotype, and a growing body of evidence supports the robust prediction of FC for AD. Our approach integrates these insights by modeling how behavioral interventions affect FC and subsequently influence AD risk. Specifically, we train a VAE to generate FC conditioned on behavioral inputs, then use an AD prediction model to quantify changes in risk based on the altered FC. This framework allows us to infer how modifying early-life factors could influence AD susceptibility through their effect on brain connectivity. Throughout this manuscript, “FC-mediated AD risk” refers operationally to the logit generated by the ADNI-trained classifier distinguishing prevalent AD from cognitively normal status. It is not intended to represent prospective AD incidence or clinical progression.

The contributions of our work are twofold. First, although VAEs have previously been applied in causal inference settings (Louizos et al., 2017; Yang et al., 2021), to the best of our knowledge, this is the first study to use a VAE for simulating counterfactual phenotypes in silico—to estimate how hypothetical changes in exposure variables influence the phenotypes, providing a flexible and generalizable generative framework for interrogating complex variable-phenotype relationships. Second, our InterventionVAE is the first framework to explicitly model how early-life behavioral and physiological factors influence late-life AD risk mediated by brain FC, enabling quantitative estimation of how early interventions may modulate disease vulnerability. While we focus on AD and FC in this study, the framework is generalizable to other neurological conditions and neuromarker modalities.

The remainder of this paper is organized as follows. Section 2 reviews related work on lifespan data integration. Section 3 describes the InterventionVAE framework, FC-mediated AD risk model, and the datasets used, including the Human Connectome Project (HCP), UK Biobank (UKB), and Alzheimer’s Disease Neuroimaging Initiative (ADNI). Section 4 presents the results of our analyses, and Section 5 concludes with discussion and potential future extensions.

## 2 Related Work

Integrating early-life behaviors with disease risks that manifest only in old age presents a significant challenge, though it has been explored in prior work. One widely used method is MR, which uses genetic variants as instrumental variables (IVs) to proxy for exposures, offering an alternative to randomized controlled trials (Sanderson et al., 2022). MR has been used in numerous applications, including AD (Korologou-Linden et al., 2022; Su et al., 2023), schizophrenia (Veeneman et al., 2022), and cancer (Wade et al., 2022). For example, one recent study employed MR to assess the influence of 12 modifiable lifestyle factors such as lipid levels, cholesterol, alcohol consumption, and smoking on AD risk (European Alzheimer’s & Dementia Biobank Mendelian Randomization (EADB-MR) Collaboration et al., 2023). Most of these factors did not show statistically significant associations with AD risk, except for High Density Lipoprotein and ApoE genotype. We attribute this, in part, to the weak instrument problem: it is often difficult to identify genetic variants that are strongly associated with complex behaviors like smoking (Andrews et al., 2019). Additionally, issues such as pleiotropy and violations of the gene-environment equivalence assumption further limit the reliability of MR for certain lifestyle exposures (Sanderson et al., 2022; VanderWeele et al., 2014). In contrast, our current study focuses only on behavioral and lifestyle factors that show significant correlations with brain FC (see Supplementary Materials). Behaviors that do not explain variance in FC are effectively neutralized during inference, with their influence on downstream outcomes averaging to near zero.

Another important line of related research involves mediation analysis, which typically decomposes the impact from an exposure to an outcome into direct and indirect components via a mediator (Rijnhart et al., 2021). Previous studies have used FC between specific brain regions as mediators for cognitive ability in MCI (Huang et al., 2024). More recently, high-dimensional mediation analysis techniques, including those based on Bayesian methods and deep learning, have enabled the use of entire FC networks as mediators (Nath et al., 2023; Tian et al., 2024). Some studies have also examined how FC mediates the relationship between genetic factors and disease outcomes (Dai & Zhang, 2024). However, a critical limitation of classical mediation analysis in the context of our research is that it requires all three components, that is, exposure, mediator, and outcome, to be measured within the same individual. This requirement is impractical when studying how early-life behaviors impact late-life outcomes like AD, due to the decades-long temporal gap and data availability constraints. In contrast, our method overcomes this constraint by leveraging separately collected population datasets under different life stages, deriving generative modeling to infer plausible mediating trajectories and estimate causal effects across lifespan-separated variables.

A core part of our method makes use of a VAE. The VAE is a deep generative latent-variable model that learns a probability distribution from which it can sample new data points (Kingma & Welling, 2019). VAEs have been widely adopted in medical research for purposes such as generating synthetic images for data augmentation (Rais et al., 2024), dimensionality reduction, and learning interpretable latent spaces (Doncevic & Herrmann, 2023; G. Kim & Chun, 2023). Notably, VAEs can also model causal effects by accounting for unobserved confounders (H. Kim et al., 2021; Louizos et al., 2017). This property is advantageous in our interventional VAE model: covariates not explicitly conditioned on are nonetheless captured in the latent distribution, mitigating confounding bias.

Several important extensions of the basic VAE framework have been proposed, including the conditional VAE (cVAE) (Sohn et al., 2015), the vector-quantized VAE (VQ-VAE) (van den Oord et al., 2018), and contrastive VAEs (Aglinskas et al., 2022). Among these, the cVAE is particularly relevant, as it augments the original VAE formulation by incorporating auxiliary conditioning variables, enabling the generation of data conditioned on labels or other contextual information. The proposed intervention VAE can be viewed as an extension of the cVAE, with additional mechanisms designed to decorrelate latent features from phenotype variables and to provide a simpler yet more robust approach to managing the multidimensional latent space. Although the VQ-VAE introduces a discrete latent representation that improves reconstruction fidelity in some domains, such discretization offers limited interpretability in the context of feature-space alterations induced by interventions. Similarly, contrastive VAEs were not adopted here, as our UKB and HCP datasets already contain explicit labels for all FC input features, rendering the additional complexity of unsupervised contrastive learning unnecessary for this first-of-its-kind study.

From a neurobiological perspective, several recent advances in VAE have applications to neuroscience and brain modeling, including studies exploring improved neural interpretability, causal latent representations, and biologically informed generative architectures. For instance, Roy et al. (2026) introduced a causal-interventional VAE for mechanistic interpretability in neural systems, while Jeffares and Liu (2025) and Xu et al. (2025) proposed VAE formulations addressing discrete neural representations and likelihood-free inference, respectively. Additionally, Wu et al. (2025) developed Koopman–Kalman VAEs for modeling neural time series dynamics, and Bhadwal et al. (2025) investigated stability and posterior collapse mitigation in brain-inspired VAE frameworks. While these studies contribute valuable methodological progress at the intersection of deep generative modeling and neuroscience, none directly implement the intervention-oriented latent modeling and counterfactual inference that define our approach.

Finally, a substantial body of research exists on the integration of multimodal data, such as multi-omics (Ballard et al., 2024; T. Wang et al., 2021; Zheng et al., 2024), or imaging with omics data (Paverd et al., 2024; Watson et al., 2021), including in the context of fMRI and brain-related gene expression in AD studies (Chan et al., 2022; Povala et al., 2024; Yoon et al., 2024). Other studies have combined multiple imaging modalities to improve predictive performance (Qu et al., 2025). However, these approaches primarily integrate data “horizontally” by combining subjects from different datasets into a larger aggregated dataset, which is fundamentally different from our objective of linking subjects from different databases measured under various life stages “vertically” to mimic different information collection at a subject-level. Our approach offers a novel method for integrating temporally and biologically disparate data into a coherent, predictive framework.

In our study, “horizontal” integration refers to the most common practice in data integration by pooling subjects from one or more datasets into a single enlarged cohort and then learning group-level patterns. This strategy increases sample size and statistical power, but it primarily treats each observation as an independent cross-sectional collection. As a result, it is well-suited for identifying stage-specific signatures, yet it is not designed to “collect” subject-level information across the lifespan, as it does not explicitly link observations corresponding to different life/disease stages into a coherent per-subject trajectory. By contrast, “vertical” integration aims to connect information across stages so that, even when longitudinal follow-up is unavailable, one can approximate the kind of multi-stage information that would ordinarily be collected within the same individual. This is exactly the setting we face: available datasets often capture different populations across younger CN, older CN and AD, and are acquired under heterogeneous protocols. Our study achieves this “subject-level” integration through the generative, intervention-aware design of InterventionVAE. Rather than requiring repeated scans of the same person, the model learns a subject-specific latent representation of each individual’s FC and a phenotype-conditioned decoder, enabling FC to be generated conditionally on demographic, genetic, lifestyle, and physiological factors.

## 3 Materials and Methods

This section describes the structure of the lifestyle factor-conditioned InterventionVAE for generation of FC data, the datasets used to train the VAE, the dataset used for the AD risk model, and the experiments to intervene on modifiable lifestyle factors and quantify the resulting change in AD risk. We first outline the structure of the VAE used for intervention analysis. We then describe three datasets used for our investigation of early-life risk factors for AD. The first of these is the ADNI dataset of AD patients as well as age-matched cognitively normal controls, used to create our FC-based model of AD. The second dataset is the UKB cohort of older but still largely cognitively normal adults, containing both neuroimaging data and a large dataset of behaviors and physiological risk factors for each subject. The last one is the HCP dataset of young adults with additional behavioral and physiological risk factors, used to identify AD risk in an early-life stage. Preliminary simulation experiments to test our model’s ability to identify causal changes in risk in the presence of a known ground truth are given in the Supplementary Materials. Additionally, in the present study, cohort and acquisition protocol are closely aligned with differences in age range, disease composition, and overall study design. Consequently, scanner-related variation cannot be cleanly separated from genuine population-level differences, and applying cross-cohort harmonization could remove meaningful biological variation together with acquisition effects. We have, therefore, not applied ComBat across ADNI, UK Biobank, and HCP, but we now explicitly acknowledge the absence of established cross-cohort measurement invariance and transportability as an important limitation.

### 3.1 Variational autoencoder for intervention analysis

An overview of our proposed use of a VAE for AD risk quantification is shown in Figure 2. The idea is to condition a VAE on, in our case, non-modifiable/genetic factors, modifiable risk factors, and physiological measurements in order to synthetically generate FC consistent with intervention on those factors. By training a generative model, we can vary one particular risk factor while holding all others constant, thereby disentangling the effects of each modifiable or non-modifiable risk factor.

**Fig. 2.**
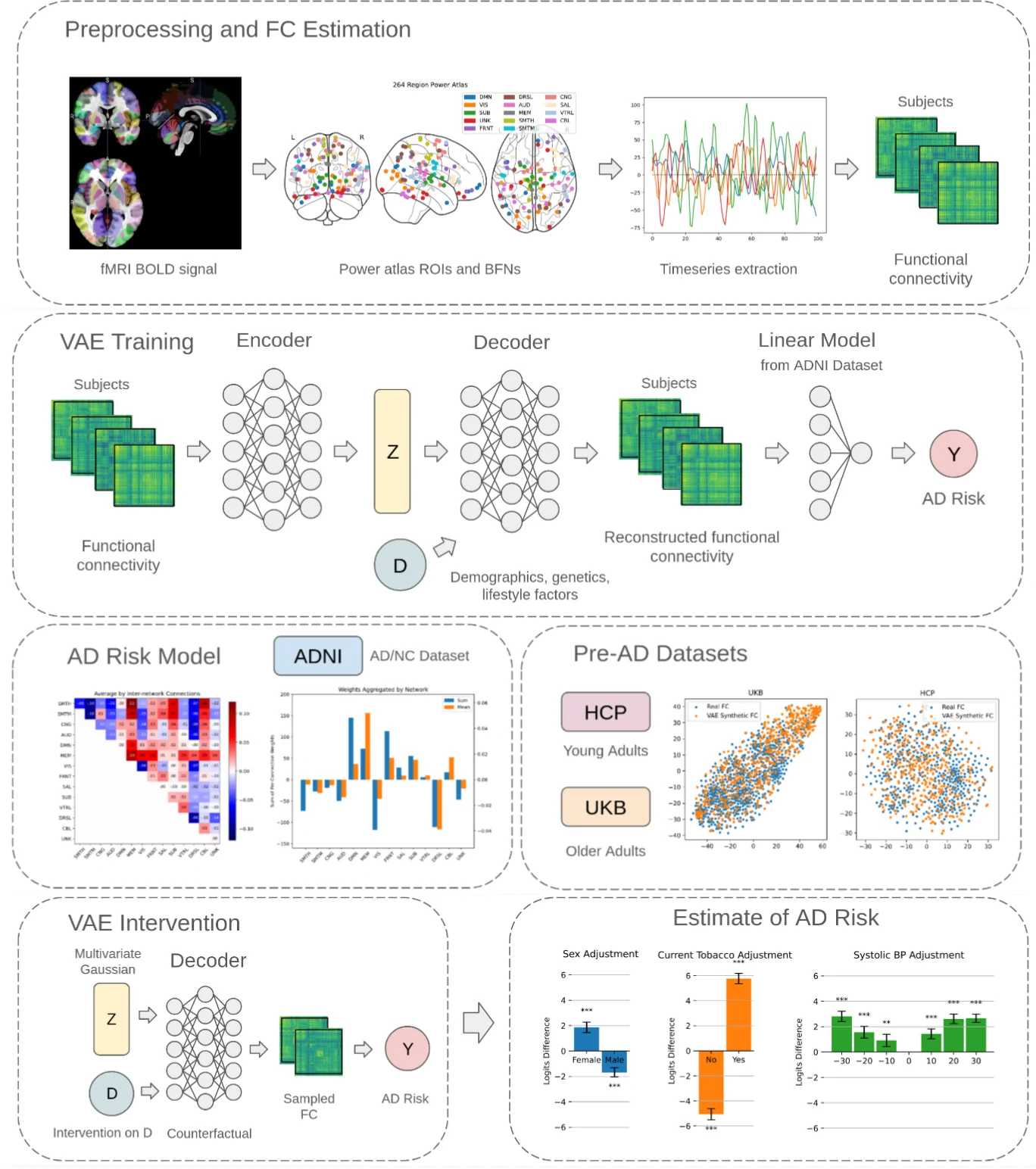
An overview of our proposed VAE-based model for intervention on demographic, genetic, and early-life lifestyle factors D to quantify AD risk mediated by FC. An AD risk model is created using the ADNI dataset and risk is evaluated on the HCP and UKB datasets. A VAE consisting of an encoder and decoder reconstructs FC from a latent space that has a target distribution of a multivariate Gaussian. The decoder is conditioned upon factors D and the latent state Z is made to be uncorrelated with D during training. During counterfactual intervention, the change in AD risk is measured for a particular intervention on D.

#### 3.1.1 Structure of the InterventionVAE

An autoencoder (AE) converts raw features into a lower-dimensional latent space via a learned encoder function $\text{z}=E_{\phi }\left(\text{x}\right)$, along with a decoder function to convert the latent features back into a reconstructed version of the input $\hat{\text{x}}=D_{\theta }\left(\text{z}\right)$. The AE is trained to minimize the difference between the reconstruction $\hat{x}$ and original input x, where in our study x is the vectorized subject FC. By contrast, a VAE trains the encoder function $E_{\phi }\left(x\right)$ to produce latent features that approximate a known probability distribution $p_{\theta }\left(z\right)$, most often taken to be a standard multivariate Gaussian distribution $p_{\theta }\left(z\right)=N\left(0,I\right)$ (Kingma & Welling, 2022). This allows for artificially constructing latent samples $z_{\text{samp}}$ from the approximated distribution, followed by the conversion of those latent features to samples of the original distribution $p_{\theta }(\text{x}|\text{z})$ by passing through the decoder function ${\text{x}}_{\text{samp}}=D_{\theta }\left({\text{z}}_{\text{samp}}\right)$. A VAE is trained by maximizing the Evidence Lower Bound (ELBO). Maximizing the ELBO is equivalent to maximizing the reconstruction probability $\text{log}\;p_{\theta }(x|z)$ while minimizing the KL divergence between our empirical and target distributions, most often the standard Gaussian distribution mentioned previously.

While prior research has explored conditioning VAE models on user-specified inputs (Razavi et al., 2019; Sohn et al., 2015), and practical applications have demonstrated zero-shot generation capabilities (Ramesh et al., 2021), these approaches typically lack explicit consideration of subject-specific phenotypes. Similarly, VAEs have been employed to generate synthetic FC data for data augmentation (J.-H. Kim et al., 2021), but often without integrating phenotype information. In contrast, our model introduces a novel phenotype-aware conditioning mechanism, enabling the generation of synthetic FC data that more accurately reflects individual variability and improves downstream analysis. In our InterventionVAE model, the observed factors of interest, such as age and sex, are not intended to be represented by the latent variables. Instead, they are provided explicitly as conditioning inputs to the decoder so they can be manipulated directly for intervention and counterfactual analysis. The latent code is designed to capture the remaining FC variability that is not explained by these conditioned factors, including unobserved influences and residual inter-subject differences needed for accurate reconstruction. Because of this design, we do not expect the latent space to organize strongly by age or sex in the way one might see in an unconditional representation-learning model. The latent distribution is regularized toward an isotropic Gaussian so that sampling from it produces realistic synthetic FC.

In this work, we include phenotypic features as input to the decoder function $\hat{x}=D_{\theta }\left(z,d\right)$, where z are the latent features and d are the subject phenotypes. During training, we decorrelate the latent state $z=E_{\phi }\left(x\right)$ from phenotypes features d so that all of the FC signal that can be attributed to phenotypes is based on user-provided input and not on the encoded latent features. To this end, we make several modifications to the traditional VAE loss function.

#### 3.1.2 Incorporate phenotype information

First, the term representing reconstruction error in the loss function remains unchanged with regard to the ELBO formulation, except for the introduction of phenotype information:

ℒRecon=1N∑i=1N||xi−Dθ(zi,di)||22, (1)

here N is the number of subjects, ${\text{x}}_{i}$ are the input features (vectorized FC), ${\text{z}}_{i}=E_{\phi }\left({\text{x}}_{i}\right)$ are the latent features derived from the encoder, and ${\text{d}}_{i}$ are subject i’s phenotypes.

#### 3.1.3 Extension to multidimensional latent space

Second, when considering a multivariate standard normal distribution $p_{\theta }\left(\text{z}\right)=N\left(0,\text{I}\right)$ for the latent features, the KL divergence part takes the form (K. P. Murphy, 2023):

$$\begin{array}{l} D_{\text{KL}}\left(N\left(\mu_{z},\Sigma_{z}\right)∥N\left(\text{0},\text{I}\right)\right)=\\ \;\frac{1}{2}\left[\text{tr}\left(\Sigma_{z}\right)+\mu_{z}^{⊤}\mu_{z}-\text{log}\left(\text{det}\left(\Sigma_{z}\right)\right)\right], \end{array}$$ (2)

which is challenging because it requires computing and backpropagating through the log-determinant of the empirical latent covariance matrix $\Sigma_{z}$.

We note that the ELBO loss function of the standard VAE is applicable to scalar latent features *z* and not multi-dimensional latent features $z\in {\mathbb{R}}^{N_{z}}$. This may allow for a non-diagonal covariance matrix in the empirical distribution of latent features $q_{\phi }(\text{z}|\text{x})=N\left(0,\Sigma \right)$. Thus, we modify a part of the ELBO loss function to specifically target a diagonal covariance matrix and zero expected value for the latent features:

$$\begin{array}{l} {ℒ}_{Cov}=\frac{1}{N}||ZZ^{T}-NI|{|}_{F}^{2}\\ {ℒ}_{Mean}=\frac{1}{NN_{z}}\sum_{i=1}^{N_{z}}||\mu_{z_{i}}|{|}_{2}^{2}, \end{array}$$ (3)

where $\text{Z}\in {\mathbb{R}}^{N_{z}\times N}$ is the Gram matrix of all $N_{z}$ latent features for all N subjects, ${\text{z}}_{i}$ is the vector of latent feature i for all N subjects, and $\mu_{z_{i}}$ is its mean.

#### 3.1.4 Decorrelate latent features from phenotypes

Third, we introduce a term that penalizes correlations between the empirical latent features and the modifiable, non-modifiable, or physiological measurement features used to condition the InterventionVAE. We define

$${ℒ}_{\text{Decor}}=\frac{1}{N_{z}N_{d}}\sum_{j=1}^{N_{z}}\sum_{k=1}^{N_{d}}∥\rho_{z_{j},d_{k}}{∥}_{2}^{2},$$ (4)

here $\rho_{{\text{z}}_{j},{\text{d}}_{k}}$ is the empirically calculated correlation between latent feature ${\text{z}}_{j}$ and phenotypic feature ${\text{d}}_{k}$ for all N subjects.

#### 3.1.5 Classifier guidance

Finally, during training of the intervention VAE, we generate synthetic samples by randomly selecting phenotypic inputs, and penalize prediction errors relative to pre-trained models. For a single phenotypic prediction derived from a synthetic latent conditioned on user-specified phenotypes, ${\hat{d}}_{i}=f_{i}\left(D_{\theta }\left({\text{z}}_{\text{samp}},\text{d}\right)\right)$, we define

$${ℒ}_{Guide}=\frac{1}{N}\sum_{i=1}^{N_{d}}\left\{\begin{array}{cc} ||d_{i}-{\hat{d}}_{i}|{|}_{2}^{2} & \;d_{i}\;continuous\\ -\sum_{c}d_{i,c}\log(p_{i,c}), & d_{i}\;\text{categorical} \end{array}\right.$$ (5)

here the functions $f_{i}\left(\cdot \right)$ are models trained on ground-truth subject FC data, $d_{i,c}$ denotes the one-hot encoded true class label for phenotype i, $p_{i,c}$ represents the predicted probability for class c and phenotype i, and the loss is defined as Mean Squared Error (MSE) for continuous phenotypes and Cross-Entropy (CE) loss for categorical phenotypes

The final loss function for training the InterventionVAE is formulated as:

$$ℒ=\lambda_{1}{ℒ}_{\text{Recon}}+\lambda_{2}{ℒ}_{\text{Cov}}+\lambda_{3}{ℒ}_{\text{Mean}}+\lambda_{4}{ℒ}_{\text{Decor}}+\lambda_{5}{ℒ}_{\text{Guide}},$$ (6)

where $\lambda_{1-5}$ are the hyperparameters chosen random grid search alongside learning rate and latent dimension size.

#### 3.1.6 Inference for FC-mediated AD risk

Once trained, the InterventionVAE model is used to generate synthetic FC based on counterfactual intervention on subject phenotypes. This FC is then evaluated using an AD risk model to determine whether the intervention increased or decreased AD risk. This is modeled by

$$\begin{array}{l} y=f_{\text{AD}}\left(\text{x}\right)\\ {\text{d}}^{\prime }=\text{d}\left(do\left(d_{1}=d_{1}+d_{adj}\right)\right)\\ \text{z}\sim N\left(\text{0},\text{I}\right)\\ {\text{x}}^{\prime }=D_{\theta }\left(\text{z},{\text{d}}^{\prime }\right)\\ y^{\prime }=f_{\text{AD}}\left({\text{x}}^{\prime }\right)\\ \Delta =y^{\prime }-y, \end{array}$$ (7)

where d are the phenotypic factors, ${\text{d}}^{\prime }=\text{d}(do(d_{1}=d_{1}+$ $d_{adj})$ are the phenotypic factors after intervention or adjustment, z is a sample from an isotropic multivariate normal distribution, ${\text{x}}^{\prime }$ is a single subject’s sampled synthetic FC conditioned on $d^{\prime }$ , and $y^{\prime }$ is the quantified FC-mediated AD risk for the subject with phenotypic factors ${\text{d}}^{\prime }$, estimated from ${\text{x}}^{\prime }$ using risk model $f_{\text{AD}}$ . This is compared with the original FC-mediated AD risk y estimated from the original subject FC x to derive the logits change Δ due to the intervention, averaged over the entire cohort.

The VAE model was trained on a Linux workstation with 64 GB of RAM and an Nvidia RTX 3060 graphics card. The hyperparameters used for training were $\lambda_{\text{Recon}}=1,000$ , $\lambda_{\text{Cov}}=10,000$ , $\lambda_{\text{Mean}}=1$ , $\lambda_{\text{Decor}}=10$ , and $\lambda_{\text{Guide}}=100$ . Additionally, classifier guidance models used the scikit-learn Ridge regressor with α=100 for continuous variables and scikit-learn LogisticRegression classifier with C=100 for categorical variables (Pedregosa et al., 2011). The encoder/decoder layers had size equal to input feature dimension of the vectorized Power atlas FC, that is, 34,716. The VAE had two hidden layers of size 120, and a latent dimension of 100. We note that because of the unique nature of the InterventionVAE model, the encoder/decoder layers are not symmetric as in a standard conditional VAE: the encoder layer is fed with FC data but without covariate/risk factor data, while the decoder layer is specifically conditioned upon risk factor features. On the other hand, the latent space that is created by the encoder is specifically decorrelated from risk factor/covariate data during training. This is not a standard part of the conditional VAE model and can be seen as specific to the InterventionVAE model. The Supplementary Materials contain a section on the ablation of the decorrelation loss during training with known ground-truth synthetic data which highlights the importance of the decorrelation loss as well as the prediction loss of the InterventionVAE model during training.

Training was performed using the PyTorch Adam optimizer with a learning rate of $5\;\cdot \;10^{-5}$ and a weight decay of 0, with 10,000 training epochs and no early termination. Hyperparameters were chosen using random grid search based on how well the synthetic samples created using the VAE recapitulated group differences in the original FC data.

### 3.2 ADNI dataset and risk predictive model

The ADNI (https://adni.loni.usc.edu) is an ongoing study with the primary goal to determine whether clinical, imaging, genetic, and biochemical biomarkers can be combined to understand the pathology of AD and track the progression of the disease at an early stage. Note that by AD pathology we specifically refer to AD-related functional alterations, AD status, or FC-based predicted AD risk. In our current study, we focus on subjects with acquired fMRI scans, and 699 subjects were included with an average age of 72.59±6.8 years. Scanning was conducted at multiple sites using GE, Philips, and Siemens scanners. The TR was variable and ranged from 6 to 0.6 seconds depending on scanner, subject, and date of scan. Coregistration and warping to MNI space of resting-state fMRI scans was carried out using SPM12 (Friston, 2007). BOLD signal was extracted using the 264 ROI Power atlas (Power et al., 2011), and the resulting timeseries were bandpass filtered between 0.01 and 0.15 Hz or the highest frequency available in the data to eliminate scanner drift, noise, heartbeat, and breathing signal. Pearson correlation between pairs of ROIs were taken to create per-subject FC maps.

The Power atlas was selected due to its high spatial resolution and balanced representation of major functional networks, including default mode, frontoparietal, dorsal and ventral attention, somatomotor, visual, and subcortical systems. This network-based organization enables more fine-grained and functionally meaningful assessments of large-scale connectivity patterns than coarser anatomical parcellations. Importantly, previous studies have demonstrated that the specific choice of parcellation scheme has a minimal effect on overall functional connectivity patterns, as long as the atlas provides adequate whole-brain coverage and a reasonable number of ROIs (e.g., Arslan et al., 2018; Eickhoff et al., 2018; Gordon et al., 2016). Thus, using the Power atlas allows comparability with a large body of existing literature while maintaining generalizability across parcellation frameworks.

A total of 737 scans consisting of scans of subjects with current AD diagnosis or current cognitively normal status were processed. ADNI subjects were selected based on their fMRI scans successfully completing our SPM12-based processing pipeline. Participants exhibiting excessive head motion during fMRI acquisition were excluded from analysis. Motion thresholds were defined a priori as translational displacements exceeding 3 mm or rotational displacements greater than 3 degrees in any direction. Subjects whose motion exceeded these limits in more than 20% of volumes were removed from the dataset to ensure data quality and minimize motion-related artifacts. The version of the ADNI data that we had access to was enriched in cognitively normal subjects, and we further limited subject inclusion by retaining only a single scan per subject, whether cognitively normal or having an AD diagnosis. Scans of subjects corresponding to mild cognitive impairment were excluded.

The remaining scans were divided into 87 AD male, 68 AD female, 235 cognitively normal male, and 347 cognitively normal female scans. In order to avoid the confounding effect of sex on AD diagnosis present in this dataset (relatively more AD males compared to AD females, and relatively more cognitively normal AD females compared to males), a balanced dataset was created with randomly selected 68 subject scans from each category, for a total of 272 scans. There was no overlap between subjects, that is, we did not include subjects who started as CN and progressed to AD. A linear logistic regression model was then trained using the scikit-learn package (Pedregosa et al., 2011) to predict AD from the scans of these 272 subjects. Subjects were then randomized and the learning process repeated for 100 bootstrap repetitions. Finally, AD-predicting FC model weights from all logistic regression models were averaged to create a de-biased model that was representative of the whole subset of the ADNI dataset used in this work. Details of the bootstrap procedure may be found in Supplementary Section “Impact of Bootstrap Repetition Number on AD Risk Model.”

Concerning the ability of FC to provide information related to AD risk, we believe FC holds substantial potential as a biomarker in AD diagnostics and classification, and recent studies indeed report extraordinary accuracy when fully exploiting FC features. For example, in one early rs-fMRI study combining graph-theory metrics of FC, the authors achieved 95% in distinguishing AD patients from healthy controls using selected network features and machine learning (Khazaee et al., 2015). In another work, a systematic review found that rs-fMRI-based ML models achieved an average binary classification accuracy of 0.99 (95% CI 0.34–1.00) for AD versus controls, with reported specificity up to 0.98 and sensitivity up to 0.94 (C. Wang et al., 2025). Further, a review of rs-fMRI FC analyses lists studies obtaining 97.5% accuracy for AD versus normal controls classification using connectivity latent features and SVM (Alarjani & Almarri, 2024). We, thus, feel confident in our use of FC as a surrogate biomarker for AD in our intervention VAE-based counterfactual study.

### 3.3 UKB dataset

The UKB is a large-scale biomedical database from over 500,000 participants, primarily collected during mid-to-late adulthood. Resting-state fMRI data as well as demographics, lifestyle factors, and physiological measurements were collected for 40,623 UKB participants. A fraction of UKB subjects had both a first and second fMRI scan, but only a single scan, the first, was used to train the InterventionVAE model. Correlation between 124 fields, consisting of questionnaire fields, demographics, and physiological measurements, was performed with resting-state fMRI-derived FC. A set of 20 fields significantly correlated with FC were selected for inclusion in the VAE model and further analysis. These fields and their summary statistics are given in Supplementary Table S5. A table of all correlations including field ID and description included in the preliminary investigation of fields associated with FC change is provided in the Supplementary Materials.

Only those subjects that had both a resting-state fMRI scan and values for all fields were retained in the analysis, leading to the inclusion of 29,466 subjects with average age 64.9±7.5 years. UKB resting-state fMRI was acquired at a resolution of 2.4 by 2.4 by 2.4 mm with an 88 by 88 by 64 FOV matrix. The duration of the scan was 6 minutes, using a TR of 0.735 seconds, resulting in 490 timepoints per scan. The TE was 39 ms, and the scanner protocol used GE-EPI with x8 multisclice acceleration, no iPAT, a flip angle of 52°, and fat saturation (Alfaro-Almagro et al., 2018; Miller et al., 2016; Smith et al., 2022).

We preprocessed the original resting-state fMRI scan data using SPM12, including scan volume coregistration and warping to MNI space (Friston, 2007). The BOLD signal was then extracted from 264 ROIs defined in the Power atlas (Power et al., 2011) to create timeseries at each ROI. These timeseries were bandpass filtered between 0.01 and 0.15 Hz to remove scanner drift, noise, heartbeat, and signal associated with breathing. The Power atlas was used, consistent with the AD risk model developed using the ADNI dataset. The Pearson correlation between each pair of ROIs was then taken to create FC maps for each subject. These FC maps along with the 20 data fields extracted earlier were then used in the following VAE-based intervention analysis. For reference, the brain functional networks making up the Power atlas and the distribution of ROIs therein are given in Supplementary Table S8.

### 3.4 HCP dataset

In addition to the UKB dataset, we tested our InterventionVAE model in the HCP young adult dataset (Van Essen et al., 2012). This dataset gives insight into AD-associated FC changes in a population before that population is of an age to show clinical signs of AD or MCI. Resting-state fMRI as well as physiological measurements were acquired for 456 subjects from the HCP young adult dataset, aged 28.5±3.69 years. The 13 fields selected for inclusion into the VAE-based analysis are listed in Supplementary Table S6. These were chosen in order to correspond most closely to the 20 chosen fields in the UKB dataset listed in Supplementary Table S5, although, since the data collection for HCP was not as thorough as for UKB, not all UKB fields had an analogue in the HCP dataset.

Resting-state fMRI for the HCP dataset was acquired using a Siemens Skyra 3T scanner using 3 mm isotropic resolution at a TR of 0.7 seconds (Uğurbil et al., 2013). The scan time of 29 minutes resulted in subjects with an fMRI time course of 1,200 time points. Temporal data were bandpass filtered between 0.01 and 0.15 Hz to eliminate scanner drift, noise, heartbeat, and breathing signal. Coregistration and warping to MNI space were performed using SPM12 (Friston, 2007). As for the UKB data, BOLD signal was extracted using the 264 ROI Power template (Power et al., 2011), and the Pearson correlation between pairs of ROIs was used to create per subject FC maps. The HCP 500 data release was used for our study, having 493 subjects with available resting-state fMRI data. Not all of these subjects had valid values for all phenotypic fields, resulting in a final count of 456 subjects for our downstream analysis. Although the selected fields in the HCP cohort were matched closely to those in the UKB cohort, measurement units often differed (metric in UKB, imperial in HCP) and were retained as originally reported to facilitate comparisons with other studies.

Although we have many more subjects in the UKB dataset than in the HCP dataset, at most 700 subjects were used at a time to train the VAE in order to have comparable sizes for the two datasets as well as for speed of convergence. These subjects were chosen randomly from the pool of available subjects each time the VAE was trained to avoid bias. Additionally, the model was only ever conditioned on a maximum of five phenotypes at once. For the UKB dataset, these were always age, sex, waist circumference, and height, with the fifth phenotype changed based on what field was being intervened on. For the HCP dataset, these were age, sex, weight, and height, with the fifth phenotype again being variable. This was undertaken because of slow convergence/instability in training when using the whole suite of phenotypic fields.

## 4 Results

### 4.1 AD risk model

The ADNI-based model for FC-mediated AD risk is shown in Figure 3. The weights of each functional connection contributing to AD risk are shown in Figure 3A and the network-averaged weights in Figure 3B and C. It can be seen that the SMTH-SMTH, DRSL, and VIS-VIS connections^1^ are highly negative for FC-mediated AD risk. Indeed, N. A. Singh et al. (2023) has found that decreased VIS-VIS connectivity is a robust consequence of AD progression. Meanwhile, most functional connections related to the MEM network are positively correlated with AD risk, although this is less significant since fewer ROIs are part of the MEM network. Also somewhat highly correlated with increased AD risk are DMN connections. Although the mean network weights for DMN-associated connections are less positive than for the MEM network, the high number of DMN ROIs means overall the DMN network contributes the most to positive signal for AD prediction. Also notable is the FRNT network which is second behind the DMN in overall AD risk by sum of connection weights. Supplementary Table S7 shows the top 10 negatively and positively weighted functional connections for AD risk.

**Fig. 3.**
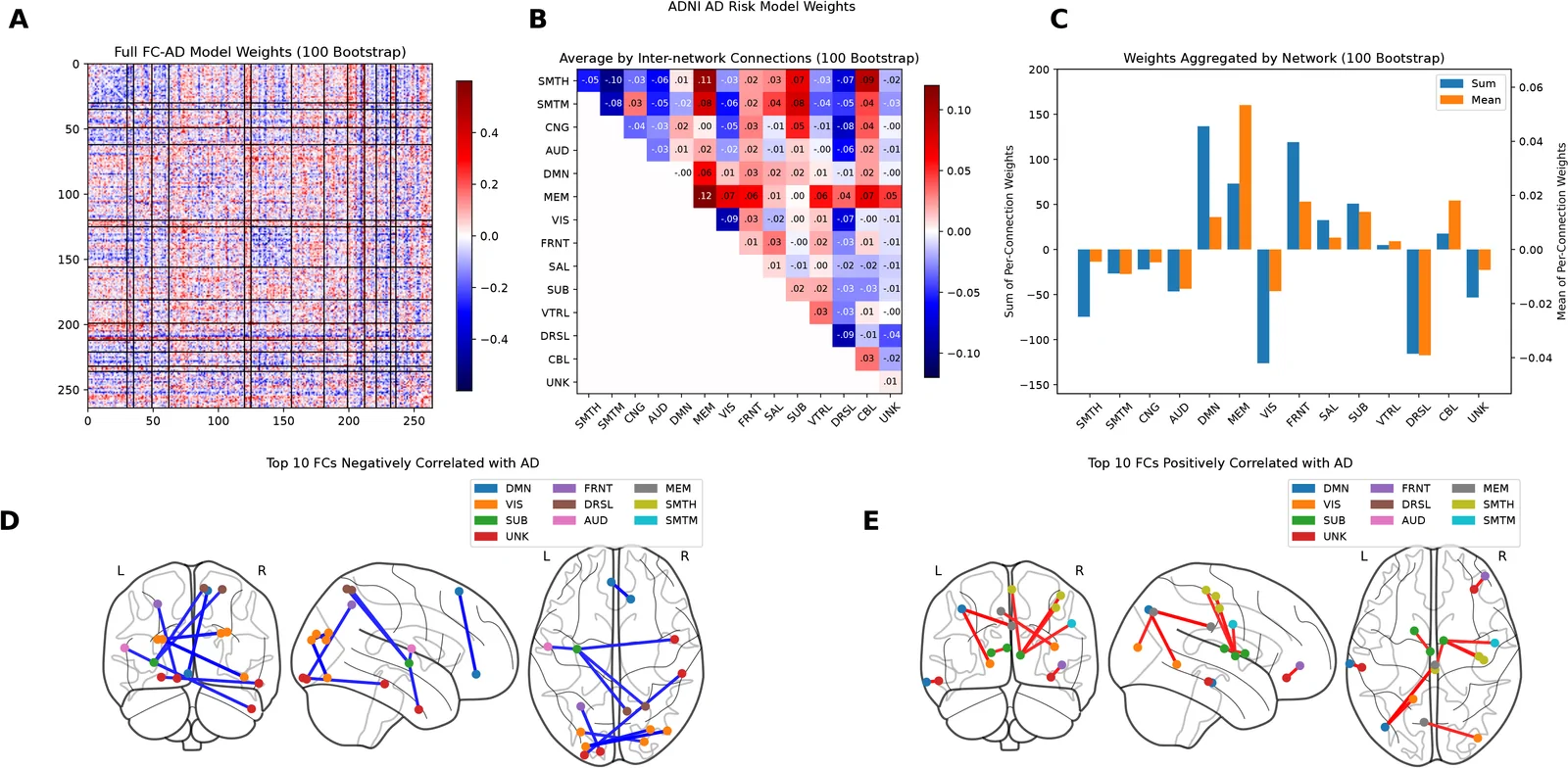
Model weights showing relationship between FC and AD diagnosis in the ADNI dataset (A). These FC-based weights for predicting AD are used to determine AD risk in the VAE-based analysis. The data are broken down by Power functional network in (B). Overall positive or negative contribution by brain functional network is shown in (C). (D) The top 10 network connections negatively correlated with AD status. Notice that most of the connections are long range and that most ROIs are part of the VIS or UNK networks. (E) The top 10 network connections most positively correlated with AD status. Note that most connection weights negatively correlated with AD are long-range connections, while weights positively correlated with AD tend to be short-range. Inter-VIS and DRSL network connections tend to be have negative weights for predicting AD. Connections involving the MEM network are especially likely to be positively correlated with AD status. Additional details of the top 10 positive and negative connections is given in Supplementary Table S7. A total of 100 bootstrap repetitions were carried out and averaged to create the FC-mediated AD risk model weights presented here.

These averaged connections weights are multiplied by subject FC, before or after VAE-based intervention, to find the logits y of AD risk, that is, $y={\text{w}}^{\text{T}}\text{x}$ , where w are the averaged connection weights from the ADNI model and x is the vectorized subject FC before or after intervention. The dimension of vectorized FC is in all cases $\text{x}\in {\mathbb{R}}^{34,716}$ , consistent with the Power atlas dimensions of 264×264 regions. Since the logits difference in AD risk is relative to the baseline value without intervention, and is dependent on the number of subjects being evaluated, we mention here that the baseline logits score without intervention was −42.6±0.8 per subject for the UKB dataset and −72.9±1.6 per subject for the HCP dataset. Any logit difference reported subsequently is higher or lower than the baseline values reported here. ADNI dataset subjects that were diagnosed with AD had an average logit score of 68.1±47.9 , and cognitively normal ADNI subjects had an average score of −52.5±50.0 . Note that in case of ADNI the standard error is standard deviation of scores among subjects while in the UKB and HCP dataset the error listed is the variance between bootstrap VAE-sampling repetitions.

### 4.2 Change in AD risk following VAE-based intervention

In this section, we describe the changes in FC-mediated AD risk following intervention on non-modifiable/genetic, modifiable lifestyle, and physiological measurement fields in both the UKB and HCP datasets. For completeness, we include intervention on factors that are not highly correlated with FC, labeled controls, in the Supplementary Materials. Specifically, Supplementary Figure S4 shows AD risk changes for factors that do not significantly change FC.

#### 4.2.1 Earlier-life risk factors leveraging UKB dataset

Figure 4 illustrates how modifiable factors and physiological measures affect AD risk via FC-mediated modeling in the UKB dataset. Tobacco use has one of the strongest effects, with current smoking increasing AD-like FC patterns; past smoking has a smaller but still significant impact, consistent with findings linking heavy smoking to dementia risk (Zhong et al., 2015). We find that UKB-provided polygenic risk scores for AD (Collister et al., 2022) are associated with higher FC-mediated AD risk, as expected.

**Fig. 4.**
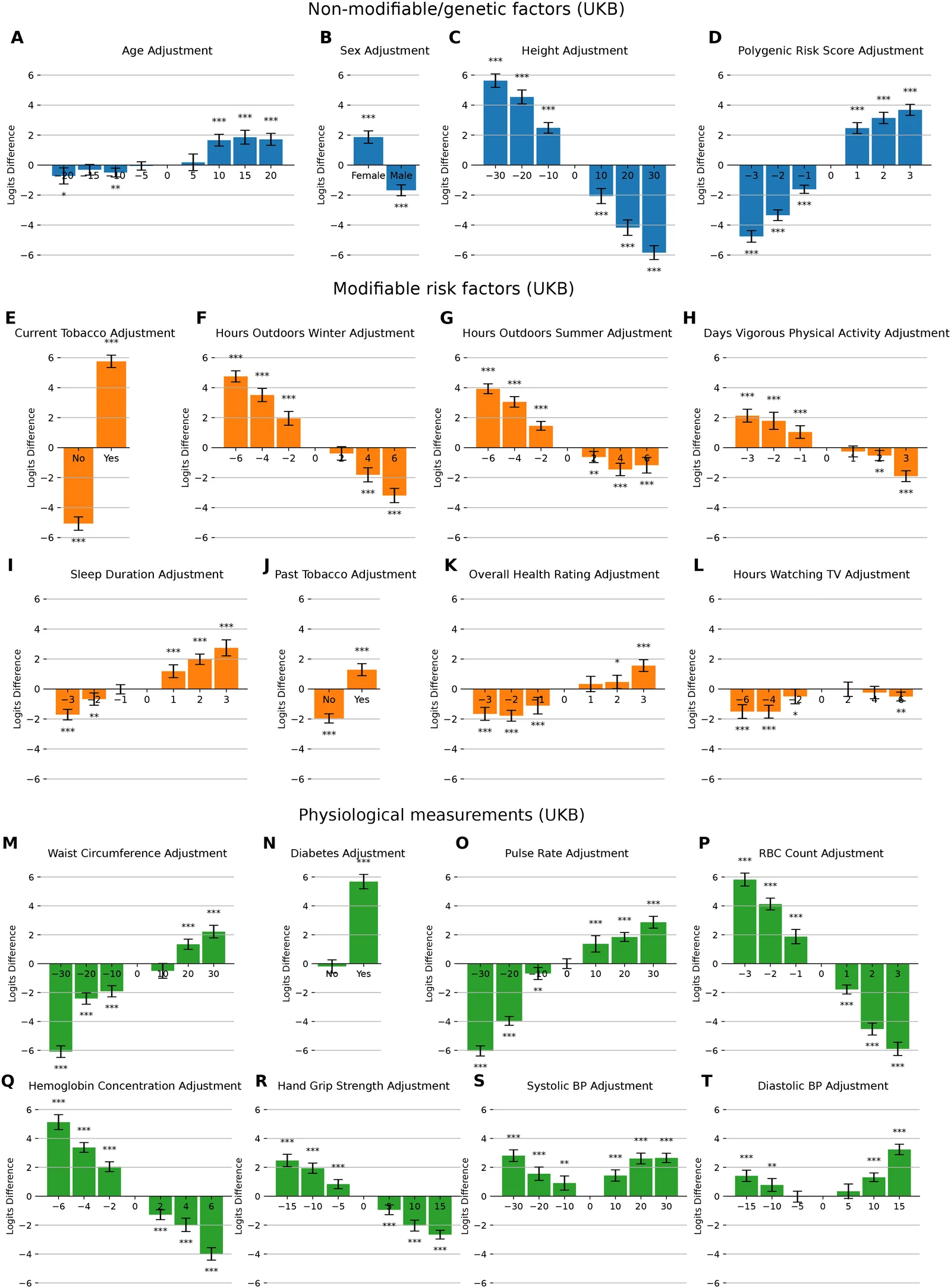
Changes in AD risk as quantified by FC mediator for non-modifiable/genetic factors, modifiable risk factors, and physiological measurements in the UKB dataset, factors labeled A–T. For each measurement, 20 bootstrap repetitions of an intervention, magnitude shown on the horizontal axis, were made and the mean and standard deviation recorded. Error bars represent the standard error, and stars represent significance level: one star (p<0.05 ), two stars (p<0.01 ), three stars (p<0.001 ). Refer to Supplementary Table S5 for summary statistics and units.

Low physical activity also raises FC-mediated AD risk, in line with studies showing exercise protects against AD through effects on amyloid β, inflammation, and blood flow (De la Rosa et al., 2020). Time spent outdoors, in both summer and winter, appears strongly protective, whereas TV viewing shows little effect. Longer sleep duration increases risk, supporting findings associating over 9 hours of sleep with doubled AD risk (Westwood et al., 2017). Better self-rated health also lowers FC-mediated risk. Note that in Figure 4K, the health rating scale is inverted, meaning a lower numerical health rating represents better health, with excellent health represented by 1 and poor health represented by 4.

Among physiological factors, higher pulse rate and diabetes diagnosis significantly increase AD risk, consistent with links between poor glucose control and earlier dementia onset (Barbiellini Amidei et al., 2021; Imahori et al., 2022; Nguyen et al., 2020; Stanciu et al., 2020). The asymmetry in diabetes intervention effects likely reflects the low prevalence of diabetes in the dataset. Increased waist circumference raises AD risk, despite lower weight often being observed in diagnosed AD patients (Hsu et al., 2016; S. Singh et al., 1988). Lower red blood cell (RBC) count and hemoglobin levels are associated with greater AD risk, matching studies that identify reduced hemoglobin as a risk factor (W.-L. Chen et al., 2022; J. W. Kim et al., 2021; Wolters et al., 2019). Interestingly, both high and low blood pressure increase AD risk, revealing a U-shaped relationship. While midlife hypertension is a known AD risk factor (Sierra, 2020), blood pressure variability and hypotension are also implicated (Ebinger et al., 2023; Landin et al., 1993; Ma et al., 2024). To our knowledge, this U-shaped pattern is a novel finding. Lastly, hand grip strength has a minor effect but trends toward a protective role, consistent with evidence linking muscle strength to reduced dementia risk (Cui et al., 2021).

#### 4.2.2 Earlier-life factors leveraging HCP dataset

Figure 5 shows FC-mediated AD risk changes from interventions on genetic, modifiable, and physiological factors in the HCP dataset. While overall trends align with the UKB analysis, notable differences emerge, especially given that HCP subjects are over 30 years younger on average.

**Fig. 5.**
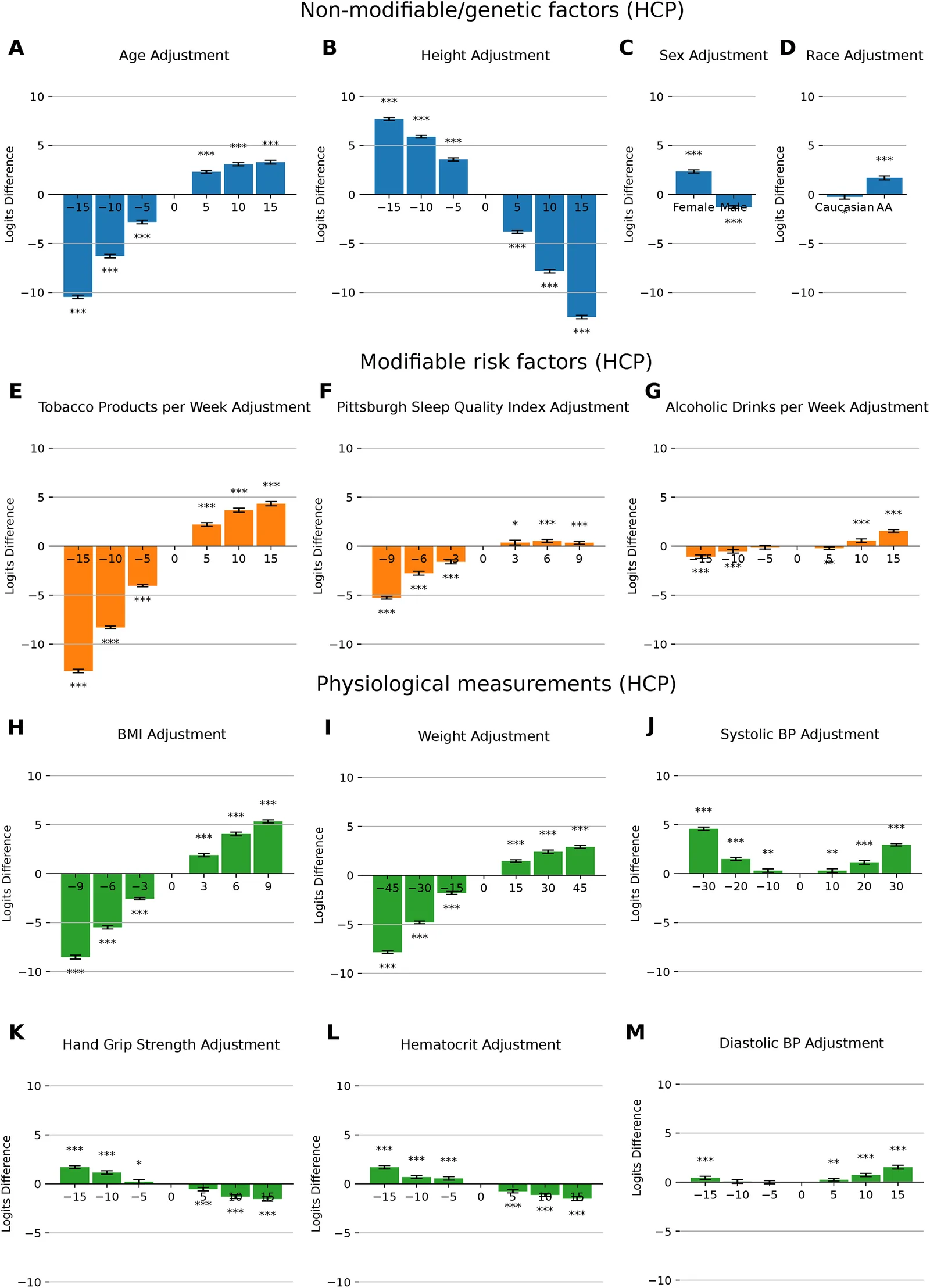
Changes in AD risk as quantified by FC mediator associated with non-modifiable/genetic factors, modifiable risk factors, and physiological measures in the HCP dataset, factors labeled A–M. Fields are ordered by magnitude of change in AD risk. See caption of Figure 4 for additional details. Refer to Supplementary Table S6 for summary statistics and units.

Age has a larger impact in HCP than UKB, likely because the same absolute age adjustment represents a greater proportional change for younger individuals. Decreasing age significantly lowers AD risk, while increasing it has a smaller effect. Height and sex show similar AD risk effects as in UKB, with shorter stature and female sex associated with higher risk—supporting genetic links between height-related pathways and neurodegeneration (Crews et al., 2010; Jørgensen et al., 2020; Larsson et al., 2017; Nelson et al., 2015). Note that in our framework, the reported effect of height is a conditional effect. When we intervene on height, we hold the other covariates fixed, including age and sex, which are explicitly included in the conditioning variables. This design reduces the possibility that the observed height effect is simply a proxy for sex or age correlations. Race also influences AD risk: African American subjects show slightly higher FC-mediated risk than Caucasians, comparable in magnitude to the male–female difference, consistent with prior findings (Barnes & Bennett, 2014).

Among modifiable factors, tobacco use again strongly increases AD risk. The HCP dataset also includes sleep quality (PSQI), revealing that better sleep is protective, though poor sleep has a less pronounced negative effect. This aligns with prior studies linking sleep disruption to AD (Gaur et al., 2022). Alcohol consumption shows minimal impact on AD risk, echoing mixed findings in the literature (Seemiller et al., 2024). In both datasets, weight and BMI show a strong relationship with AD risk, supporting findings that obesity at any age may elevate AD susceptibility (Gu et al., 2014; Whitmer et al., 2005). Especially in younger individuals, for example, the HCP dataset compared to UKB, higher weight and BMI correlate with AD-like FC patterns, suggesting an age-dependent relationship. Specifically, the same FC patterns that correlate with high BMI and weight in younger cohorts correlate with higher AD risk in our ADNI-based model. This relationship between weight/BMI and AD has been previously explored in Gu et al. (2014). Blood pressure again demonstrates a U-shaped effect, both high and low values increase AD risk, suggesting that deviation from normal ranges may be harmful even in healthy populations. Hand grip strength remains a minor but steady protective factor, consistent with its use as a biomarker for cognitive decline (Shaughnessy et al., 2020). Finally, higher hematocrit levels (as with RBC in UKB) are associated with reduced AD risk, while anemia increases risk.

### 4.3 Validation of the VAE model in recapitulating FC changes

To check the validity of synthetic samples generated by the VAE model, we tested the difference between extremes of three phenotypes in the UKB and HCP datasets: age, sex, and BMI. The resulting mean FC changes between groups for old minus young (age), male minus female (sex), and high BMI versus low BMI groups are shown in Figure 6. The synthetic samples were created using the same demographic information as real samples, with the division between groups as in Table 1. Consistent with the description of our VAE model in Section 3.1, synthetic data were obtained by training the InterventionVAE on the UKB or HCP datasets, then simulating the same number of subjects as in the real data conditioned on the same phenotype values, but sampling from the latent space instead of generating the latent space from the encoder.

**Fig. 6.**
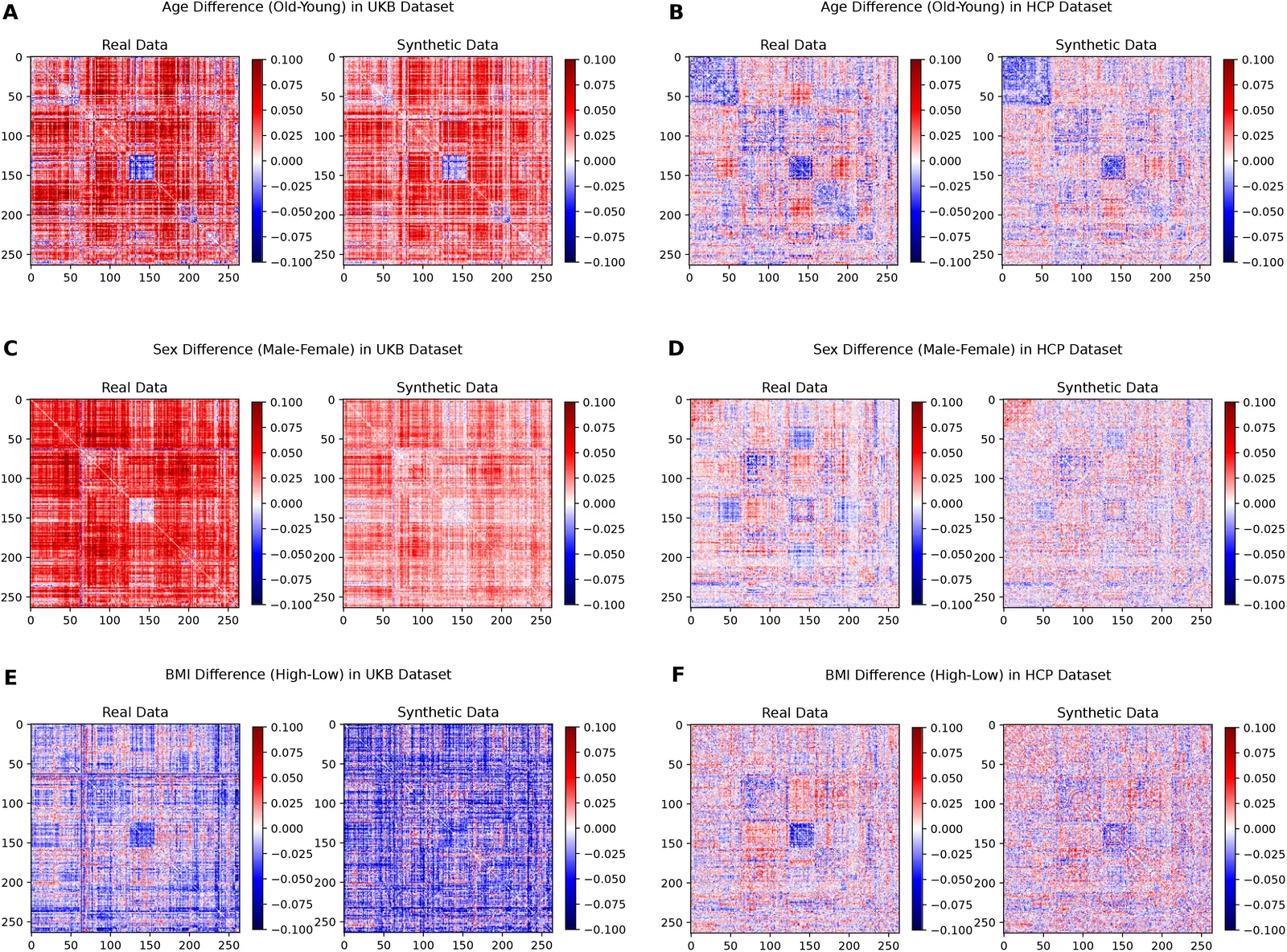
Changes in FC for real subjects groups (left of each pair) and synthetic subject groups (right of each pair) for differences in age (A, B), sex (C, D), and BMI (E, F) in the UKB and HCP datasets. We note that the synthetic, VAE-generated samples qualitatively reproduce group differences. It is also apparent that normative FC changes for the same demographic (e.g., age) are different for the UKB and HCP datasets. Values for the average FC matrices and the corresponding mean squared errors between empirical and synthetic data are reported in Supplementary Table S11.

**Table 1.** Group memberships for comparing real subject and synthetic sample group differences derived from the VAE model.

Factor	Dataset	Group 1 Criteria	Group 2 Criteria	Group 1 Size	Group 2 Size
Age	UKB	Age < 50	Age > 65	99	322
Age	HCP	Age < 25	Age > 30	82	158
Sex	UKB	Female	Male	351	349
Sex	HCP	Female	Male	249	207
BMI	UKB	BMI < 23	BMI > 28	129	235
BMI	HCP	BMI < 23	BMI > 28	179	57

The replication of group differences using synthetic samples leads us to believe the decoder accurately captures the effect of demographics or lifestyle factors on synthetically generated FC. Moreover, this highlights the main advantage of the VAE model over simply looking at group differences, that is, the ability to intervene on one specific demographic/factor and keep all others the same. This has the effect of disentangling demographics that are otherwise correlated, for example, age and height, sex and height, sex and weight, etc. The built-in disentangling effects of different demographics is one of the key benefits of using the InterventionVAE model that is not otherwise possible by using existing group differences in a naive way, due to the correlations between demographics/factors.

Finally, the similarity of distributions between synthetic FC data created via intervention by VAE and real FC data can be visualized directly by means of a low-dimensional manifold projection such as t-SNE (van der Maaten & Hinton, 2008). Figure 7 shows the t-SNE of synthetic data and real data in the UKB and HCP datasets. It can be seen that there is no discernible difference between the t-SNE projection of real versus synthetic FC data for the UKB or HCP datasets.

**Fig. 7.**
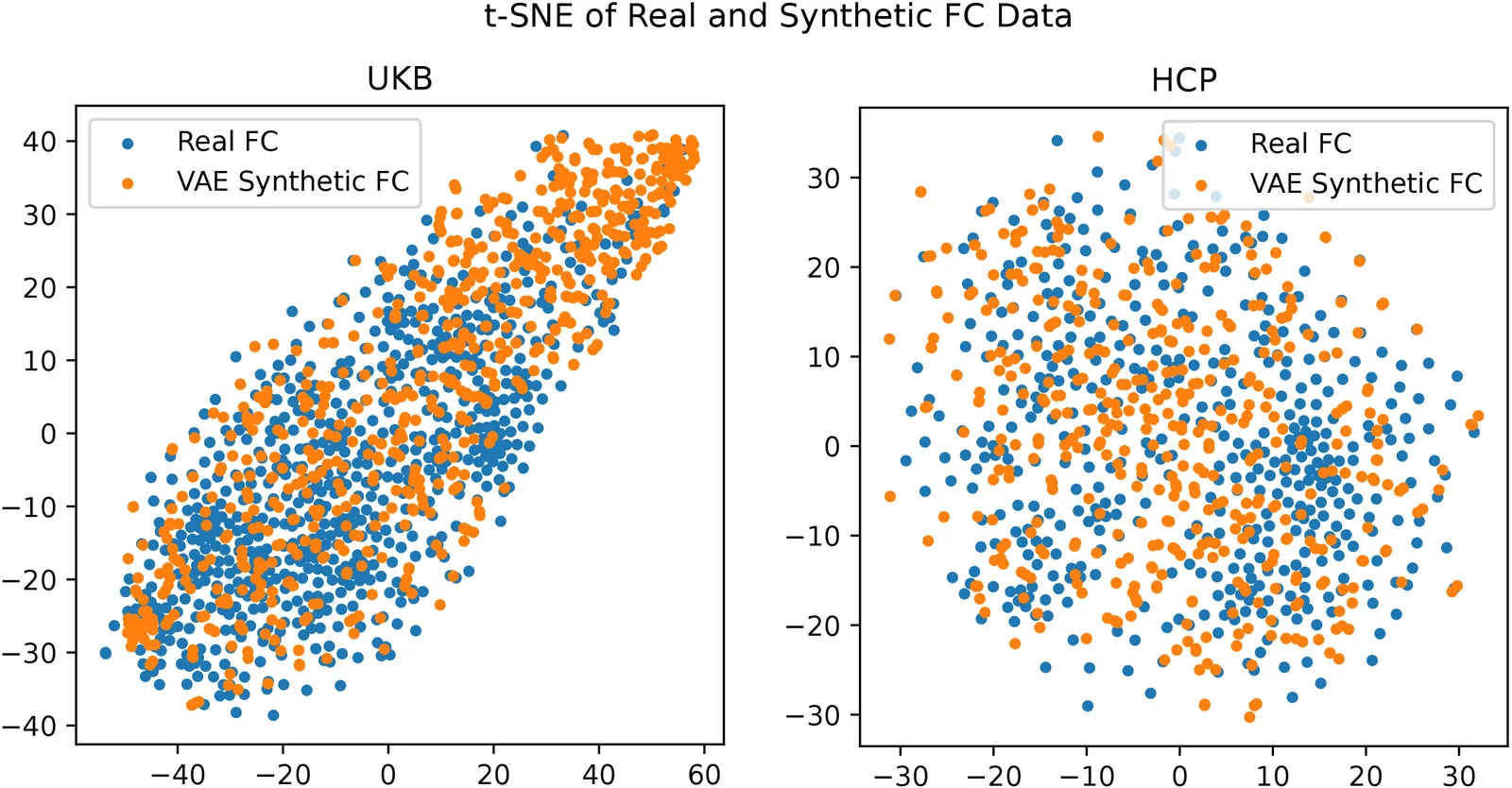
t-SNE projection of real and synthetic data from the UKB and HCP datasets. Shown are 700 subjects from the UKB and 456 subjects from the HCP datasets. Synthetic data had its latent space sampled from a multivariate normal distribution and were not a reconstruction of the original FC data. A perplexity of 10 was used to create the t-SNE.

### 4.4 Functional connections contributing to AD risk change

Understanding how interventions in the VAE model alter FC and contribute to AD risk is crucial. Figures 8 and 9 illustrate the impact of selected interventions (height, weight, waist circumference) on FC and AD risk per connection for the HCP and UKB datasets.

**Fig. 8.**
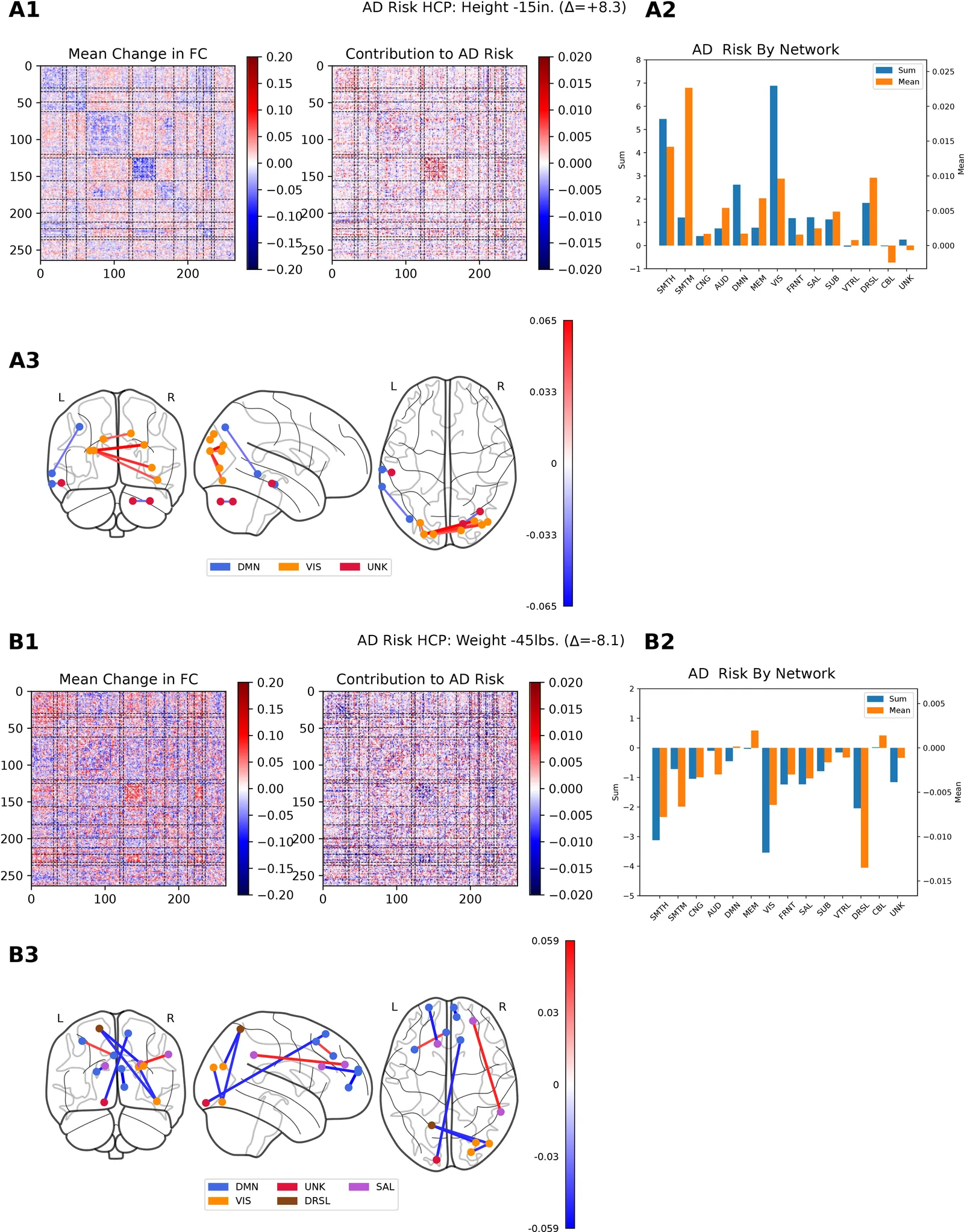
Selected changes in FC and concomitant change in AD risk profile for height (A) and weight (B) in the HCP dataset. Plots A1 and B1 show changes in FC for intervention to decrease height by 15 inches and decrease weight by 45 lbs. Plots A2 and B2 show average and total change in AD risk by brain functional network in the Power atlas. Plots A3 and B3 show most significant inter-ROI connections contributing to change in AD risk. We note that most significant connections for height are positive and in the VIS network, while connections for weight are negative and are long-range. Contribution to AD risk is calculated via the ADNI-derived model weights multiplied by average change in FC due to intervention.

**Fig. 9.**
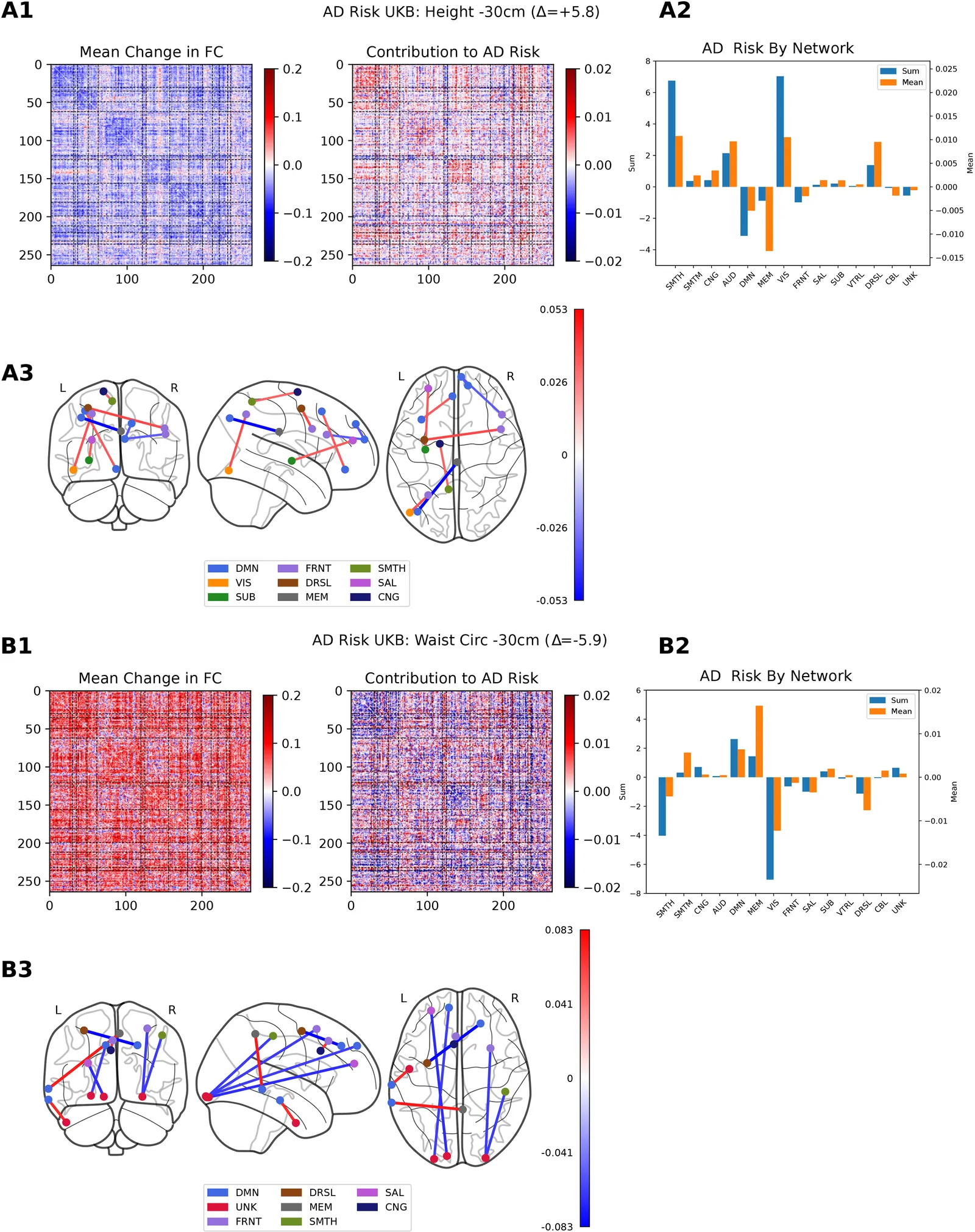
Selected changes in FC and concomitant change in AD risk profile for height (A) and waist circumference (B) intervention in the UKB dataset. Plots A1 and B1 show changes in FC for intervention to decrease height by 30 cm and decrease waist circumference by 30 cm. Plots A2 and B2 show average and total change in AD risk by brain functional network in the Power atlas. Plots A3 and B3 show most significant inter-ROI connections contributing to change in AD risk. Interventions on risk factors in the UKB cohort tend to increase or decrease FC across the whole connectome, as evident in this figure for an FC decrease in plot A1 and an FC increase in plot B1. This is in some contrast to HCP, where intervention tends to increase some inter-network connectivities while decreasing others. Contribution to AD risk is calculated via the ADNI-derived model weights multiplied by average change in FC due to intervention.

In the HCP dataset (Fig. 8), decreases in intra-network connectivity, particularly SMTH-SMTH and VIS-VIS, generally increase AD risk, while increases in these connections reduce it. SMTH-VIS and other intra-network connections tend to co-vary, but DMN-VIS shows an opposing pattern. Despite varied FC changes, each network’s contribution to AD risk is mostly unidirectional, with the exception of the CBL network (Fig. 8.A2, B2). The strongest AD risk effects come from VIS, STMH, and, to a lesser extent, DMN. Only the MEM, CBL, and UNK networks deviate from the overall risk trend, showing disagreement under certain interventions.

In contrast, the UKB dataset (Fig. 9) shows more uniform FC changes across the connectome, either largely increasing or decreasing with intervention. This is especially evident for height and waist circumference. However, the corresponding AD risk varies by network. VIS and SMTH again dominate in effect size, while DMN and MEM contribute smaller but oppositely directed effects. Unlike the HCP dataset, the UKB shows simpler global FC changes, but more diverse network-level AD risk responses.

The more uniform FC changes observed in the UKB dataset are not solely driven by age but likely emerge from multiple interacting factors. While the broader and older demographic distribution of UKB participants contributes to globally covarying FC patterns, additional sources of uniformity include the lower spatial and temporal resolution of UKB imaging data relative to HCP, as well as increased physiological and lifestyle variability that modulates global signal and vascular components of FC.

By “more uniform,” we refer to the tendency for an intervention on a single factor within the Intervention VAE to induce coherent changes in FC across multiple networks, with connections shifting collectively in the same direction. In contrast, less uniform changes indicate that an intervention produces heterogeneous effects on FC, with different networks responding in distinct ways. We believe that the more uniform FC changes observed in the UKB dataset are not solely driven by age but are likely the result of multiple interacting factors. The broader and older demographic distribution of UKB participants contributes to globally changing FC patterns, and different lifestyle and physiological variability may alter the various brain functional network components of FC.

The previous observation underscores that the FC-based VAE captures both distributed and network-specific components of functional variability. In the UKB cohort, traits such as height and waist circumference induce broad, directionally consistent FC changes—suggesting the VAE is sensitive to system-level coupling effects likely reflecting vascular and metabolic influences. Nevertheless, the model still discriminates network-dependent contributions to AD risk, with VIS and SMTH networks showing dominant effect sizes and DMN and MEM exhibiting smaller, oppositely directed effects.

Ultimately, the HCP and UKB datasets have different demographics as well as different acquisition protocols, so we should expect to encounter some quantitative differences in specific network behavior. In particular, the HCP dataset displays greater intra-network connectivity compared to the UKB. However, we see patterns of commonality, for instance in Figure 6A and B we see that while the majority of functional connectivity increases throughout the connectome in response to aging, the VIS-VIS connectivity in both the UKB and HCP dataset shows strong negative correlation with respect to aging.

## 5 Discussion

This project presents a novel framework for exploring how interventions on various phenotypic factors influence brain FC and, in turn, the risk of AD. By leveraging a phenotype-conditioned VAE, the InterventionVAE method enables counterfactual manipulation of both modifiable and non-modifiable traits to assess their impact on FC patterns associated with AD. A model of FC changes characteristic of AD was first derived using the ADNI dataset. This was followed by the development of a VAE that conditions FC on individual covariates, allowing simulations of how altering specific traits would affect FC and AD risk. Key findings revealed that factors such as height, weight/BMI, tobacco use, and age (notably in the HCP dataset) significantly influenced FC-mediated AD risk. In the UKB dataset, higher polygenic risk scores were associated with increased risk, and a surprising U-shaped relationship was observed between blood pressure and AD risk across both the UKB and HCP datasets. Beyond AD, the approach provides a flexible tool for investigating the impact of phenotypic interventions on FC in other neurological disorders, such as schizophrenia or ADHD, by adapting the underlying risk model.

While our current study focuses on resting-state fMRI, the underlying framework is adaptable to other imaging modalities. Structural MRI, for example, has been extensively used in AD diagnosis, particularly in identifying atrophy patterns in later disease stages. Applying our model to sMRI or combining it with fMRI could enable multimodal inference across disease stages. Future work may also explore task-based fMRI, which has shown improved sensitivity in predicting cognitive traits and clinical outcomes compared to resting-state data in some studies (Zhao et al., 2023). Evaluating both modalities within the same cohort could clarify their respective contributions to early AD risk modeling and assess the consistency of inferred risk trajectories.

It should be noted that our results exhibit some notable variability across the two cohorts, which is consistent with well-established differences between datasets such as UKB and HCP. These cohorts differ substantially in age distribution, sample size, scanner hardware and acquisition protocols, preprocessing pipelines, and overall population heterogeneity, all of which are known to influence FC structure. Such cohort-level factors can lead to shifts in baseline FC organization as well as differences in the apparent magnitude and spatial patterning of FC changes associated with common covariates, including sex, body mass index, and anthropometric measures (e.g., weight versus waist circumference). Given that this work is among the first to examine FC changes induced by hypothetical interventions on lifestyle and physiological factors, a complete mechanistic interpretation of all observed patterns is not yet available. Accordingly, we report the model-derived intervention effects while recognizing that both biological variability and cohort-specific characteristics likely contribute to the observed heterogeneity.

Despite this variability in FC patterns, which can be quantitatively assessed using metrics such as mean squared error, the results show meaningful convergence at the level most relevant to the present study: the predicted change in overall AD risk following intervention on each factor. Across datasets, the directionality of AD risk modulation is broadly consistent, even when the fine-grained FC alterations differ. This suggests that while cohort-specific factors may influence detailed FC responses, the higher-level conclusions regarding the relative sensitivity of AD risk to individual factors are comparatively robust to dataset heterogeneity.

Additionally, some factors examined in this framework, such as height or polygenic risk score, are inherently non-modifiable at the individual level. Interventions on these variables are therefore not intended to imply practical modifiability, but rather to support a counterfactual analysis in which individuals are compared who are identical in all respects except for a controlled variation in a single factor. In addition, certain perturbations may move individual values outside physiologically plausible or healthy ranges. The inferences presented here, however, are based on population-level average effects estimated across large cohorts rather than on individual counterfactual trajectories. Because most participants lie within normative ranges for many modifiable factors, the estimated changes in AD risk logits primarily reflect aggregate behavior across the sample. Although some individual counterfactuals may be unrealistic or extreme, the resulting average effects remain informative for characterizing group-level relationships among risk factors, FC patterns, and vulnerability to AD.

A key advantage of using a VAE architecture is its ability to encode nuisance or confounding factors within the latent space, even when they are not explicitly conditioned upon (Louizos et al., 2017). Thus, for datasets such as HCP in which site information is not modeled directly, site-related variance is still expected to be reflected in the generated FCs, albeit in an unsupervised and randomly sampled manner.

A natural extension of this work is to incorporate dynamic FC, which captures time-varying changes in brain connectivity and may better reflect the brain’s adaptive processes than static FC. Dynamic FC has shown promise in improving classification of neuropsychiatric conditions such as schizophrenia (Rashid et al., 2016), and may enhance the sensitivity of AD risk modeling to transient or subtle network disruptions. Investigating whether dynamic FC yields consistent or complementary findings compared to static FC would help deepen mechanistic insights into how early-life factors shape long-term brain function and disease susceptibility. These future directions will further validate and extend the versatility of our approach across modalities and temporal dimensions. One limitation of the current study is the lack of leave-one-out cross-validation. Imaging-site variability is a well-known challenge in multisite neuroimaging studies, and leave-one-site-out cross-validation would be valuable when site labels and sample sizes allow. In our current pipeline, however, a meaningful leave-one-site-out evaluation is limited by dataset and sample-size constraints. Site identifiers are available in ADNI, but comparable site metadata are not available in UK Biobank in a way that supports consistent site-based partitioning across the full workflow. This matters because our framework combines an AD risk model trained in ADNI with a generative model that is trained and queried using other cohorts, so a site-based generalization test is not straightforward to define across components.

In principle, validation on individual-level data, where two subjects differ in only one category, is to be desired. For a given factor, one would compare pairs of individuals who are matched on all other covariates and differ only in that factor, then ask whether the model’s predicted intervention-induced change aligns with the observed difference between the matched individuals. However, this type of validation is difficult to carry out in our setting for two reasons. First, our primary “intervention” analyses target early-life or mid-life factors using UKB and HCP, whereas ADNI provides late-life FC with diagnosis labels and is used to train the AD risk model. ADNI therefore does not contain the same breadth of early-life variables, nor does it provide the counterfactual settings needed to form well-matched pairs that differ in only one factor. Second, even when a variable is available, finding enough closely matched pairs is challenging because risk factors are correlated with each other and with age, comorbidities, and lifestyle. This would lead to very small effective sample sizes, making any individual-level paired comparison noisy and potentially misleading.

In regards to brain functional networks associated with AD, past neuroimaging studies have consistently implicated alterations said networks, with the DMN being the most extensively studied. Early resting-state fMRI work demonstrated reduced DMN activity and connectivity in AD, particularly involving the posterior cingulate cortex, precuneus, hippocampus, and medial prefrontal regions, suggesting that disruption of the DMN is closely related to cognitive impairment and AD pathology (Greicius et al., 2004; K. Wang et al., 2007). Subsequent studies extended these findings beyond the DMN, reporting alterations in the salience, attention, executive/control, sensorimotor, and limbic networks, as well as changes in connectivity between networks (Balthazar et al., 2014; P. Wang et al., 2015). More broadly, studies examining multiple intrinsic connectivity networks have reported progressive reductions in within-network connectivity across the AD spectrum, suggesting that AD-related dysfunction extends beyond individual canonical networks (Nuttall et al., 2016). Despite this growing evidence, there have been relatively limited works specifically focused on systematically identifying which functional networks are most strongly associated with Alzheimer’s disease, rather than examining predefined networks or individual connectivity alterations. This distinction is important because identifying the networks most strongly associated with AD may provide a more comprehensive characterization of disease-related functional organization and may help identify network-level biomarkers. Our own modeling mainly identifies the SMTH-SMTH, DRSL, and VIS-VIS connections, along with DMN, in which changes are indicative of higher risk for diagnosis with AD.

Finally, although the present study performs a rigorous integration of early-life behaviors and risk factors and late-life AD risk, it would be valuable to obtain a large ground-truth dataset, similar to the UKB but enrolling subjects at a younger age and following them until prodromal AD symptoms appear. Given the necessarily long time and resources required for this study, it is probably only possible with the cooperation of a national healthcare system. Even if unlikely in the near future, such a study would be the gold standard for the investigation of early-life risk factors and progression of AD in later life.

## Supplementary Material

Supplementary Material

## Data Availability

ADNI, HCP, and UKB data are available from the relevant organizations to authorized researchers. The authors do not have permission to redistribute data. Model source code is made available at https://github.com/yizekaren/intervention-vae.

## Footnotes

1 For network abbreviations, see Supplementary Table S8.
