# Supplementary material for: Intervention on modifiable lifestyle and physiological factors via variational autoencoder reveals changes in functional connectivity-mediated risk for Alzheimer’s disease
Source: Imaging Neurosci (Camb). 2026 Oct 22;4:IMAG.a.1408. doi: 10.1162/IMAG.a.1408 (PMC13736802; doi:10.1162/IMAG.a.1408)
Supplement: Supplementary file 1 [file IMAG.a.1408_supp.pdf]

# Supplementary Material

## Correlation of FC and Clinical Fields in the UKB Dataset

Prior to VAE-based intervention analysis on modifiable and physiological factors, a preliminary study was undertaken to find phenotypic fields that were significantly correlated with FC in the UKB dataset. This was accomplished in two steps. First, treating each functional connection as an independent scalar measurement for each subject, the correlation between each functional connection and phenotypic field was calculated. Next, using the resulting correlation map with FC, the dot product of each subjects vectorized FC and correlation map was produced, with a scalar value for each subject.

$$\begin{aligned} w_i &= \rho_{x_i,d} \\ m^{(j)} &= \mathbf{w}^\mathsf{T}\mathbf{x}^{(j)} \\ c &= \rho_{m,d}, \end{aligned} \tag{1}$$

Here $x_i$ is the functional connection $i$, $d$ is the phenotype of interest, $w_i = \rho_{x_i,d}$ is the correlation between the phenotype of interest and a single functional connection among all subjects, $m^{(j)}$ is the scalar dot product between the correlation map $\mathbf{w}$ and the vectorized FC $\mathbf{x}^{(j)}$ for subject $j$, and $c$ is the final correlation. The correlation between this scalar dot product and phenotypic value was then calculated and this correlation is reported in Tables 1, 2, 3, and 4.

| UKB Field ID | Description | Correlation with FC | Used |
|---|---|---|---|
| 31 | Sex | 0.282 | Yes |
| 21003 | Age at Assessment | 0.236 | Yes |
| 46 | Hand grip (left) | 0.2 | Yes |
| 47 | Hand grip (right) | 0.19 | |
| 48 | Waist circumference | 0.16 | Yes |
| 49 | Hip circumference | 0.11 | |
| 50 | Standing height | 0.21 | Yes |
| 102 | Pulse rate (automated) | 0.1 | Yes |
| 134 | Number of cancers | 0.06 | |
| 135 | Number of illness | 0.09 | |
| 136 | Number of operations | 0.04 | Control |
| 137 | Number of treatments/meds | 0.08 | |
| 738 | Household income | 0.07 | |
| 884 | Days moderate physical activity | 0.04 | |
| 894 | Activity duration (moderate) | 0.04 | |
| 904 | Days vigorous physical activity | 0.1 | Yes |
| 914 | Activity duration (vigorous) | 0.03 | |
| 924 | Usual walking pace | 0.06 | |
| 943 | Frequency stair climbing | 0.05 | |
| 971 | Frequency walking pleasure | 0.03 | |
| 981 | Duration walking pleasure | 0.04 | |
| 991 | Frequency strenuous sports | 0.05 | |
| 1011 | Frequency light DIY | 0.06 | |
| 1021 | Duration light DIY | 0.04 | |
| 1050 | Time spent outdoors summer | 0.1 | Yes |
| 1060 | Time spent outdoors winter | 0.11 | Yes |
| 1070 | Time spent watching TV | 0.09 | Yes |
| 1080 | Time spent on computer | 0.06 | |
| 1090 | Time spent driving | 0.04 | |

Table 1: Correlations between UKB fields and resting state FC based on the Power atlas.

| UKB Field ID | Description | Correlation with FC | Used |
| --- | --- | --- | --- |
| 1160 | Sleep duration | 0.11 | Yes |
| 1170 | Getting up in morning | 0.1 | |
| 1180 | Morning/evening person | 0.03 | |
| 1190 | Nap during day | 0.06 | |
| 1200 | Sleepless/insomnia | 0.05 | |
| 1210 | Snoring | 0.1 | |
| 1220 | Daytime dozing/sleeping | 0.04 | |
| 1239 | Current tobacco | 0.12 | Yes |
| 1249 | Past tobacco | 0.11 | Yes |
| 1259 | Smokers in household | 0.03 | |
| 1269 | Exposure to smoke in home | 0.02 | |
| 1279 | Exposure to smoke outside home | 0.04 | |
| 1289 | Cooked vegetable intake | 0.05 | |
| 1299 | Salad/raw vegetable intake | 0.05 | |
| 1309 | Fresh fruit intake | 0.03 | Control |
| 1319 | Dried fruit intake | 0.04 | |
| 1329 | Oily fish intake | 0.02 | Control |
| 1339 | Non-oily fish intake | 0.04 | |
| 1349 | Processed meat intake | 0.04 | |
| 1558 | Average weekly beer plus cider | 0.04 | |
| 1707 | Handedness | 0.03 | |
| 1717 | Skin color | 0.05 | |
| 1747 | Hair color | 0.08 | |
| 1767 | Adopted as child | 0.05 | |
| 1777 | Part of multiple birth | 0.04 | |
| 1787 | Maternal smoking during preg | 0.06 | |
| 1920 | Mood swings | 0.03 | |
| 1930 | Miserableness | 0.04 | |
| 1940 | Irritability | 0.03 | |
| 1950 | Sensitivity/hurt feelings | 0.02 | |
| 1960 | Fed-up feelings | 0.03 | |

Table 2: Correlation between UKB fields and resting state FC based on the Power atlas, continued.

| UKB Field ID | Description | Correlation with FC | Used |
|---|---|---|---|
| 1970 | Nervous feelings | 0.03 | |
| 1980 | Worrier/anxious feelings | 0.03 | |
| 1990 | Tense 'high strung' | 0.02 | Control |
| 2000 | Worry too long after embarrass | 0.02 | |
| 2010 | Suffer 'nerves' | 0.02 | |
| 2020 | Loneliness, isolation | 0.02 | |
| 2030 | Guilty feelings | 0.02 | |
| 2040 | Risk taking | 0.027 | |
| 2050 | Depressed 2 weeks | 0.025 | |
| 2060 | Unenthusiasm 2 weeks | 0.027 | |
| 2070 | Tenseness 2 weeks | 0.022 | |
| 2080 | Tiredness 2 weeks | 0.035 | |
| 2090 | Seen doctor for nerves | 0.031 | |
| 2100 | Seen psychiatrist for nerves | 0.028 | |
| 2178 | Overall health rating | 0.11 | Yes |
| 2188 | Long standing illness | 0.04 | |
| 2296 | Falls in last year | 0.02 | |
| 2306 | Weight change in 1 year | 0.043 | |
| 2443 | Diabetes | 0.1 | Yes |
| 2463 | Broken bones in 5 years | 0.07 | |
| 2473 | Other serious condition doctor | 0.03 | |
| 4079 | Diastolic blood pressure | 0.1 | Yes |
| 4080 | Systolic blood pressure | 0.14 | Yes |

Table 3: Correlation between UKB fields and resting state FC based on the Power atlas, continued.

| UKB Field ID | Description | Correlation with FC | Used |
|---|---|---|---|
| 21001[1] | BMI | 0.134 | |
| 21002[1] | Weight | 0.128 | |
| 23099[1] | Body fat % | 0.126 | |
| 23100[1] | Whole body fat mass | 0.131 | |
| 23101[1] | Whole body fat free mass | 0.138 | |
| 23102[1] | Whole body water mass | 0.138 | |
| 23104[1] | BMI | 0.134 | |
| 23105[1] | Basal metabolic rate | 0.124 | |
| 23106[1] | Impedance of whole body | 0.172 | |
| 26207 | PRS Alzheimer's disease | 0.09 | Yes |
| 30000 | Whole body white blood cell | 0.045 | |
| 30010 | Red blood cell count | 0.072 | Yes |
| 30020 | Hemoglobin concentration | 0.089 | Yes |
| 30080 | Platelet count | 0.032 | |
| 30520 | Potassium urine | 0.033 | |
| 30530 | Sodium urine | 0.045 | |
| 30600 | Albumin | 0.06 | |
| 30610 | Alkaline phosphatase | 0.046 | |
| 30630 | Apolipoprotein A | 0.059 | |
| 30680 | Calcium | 0.047 | |
| 30690 | Cholesterol | 0.063 | |

Table 4: Correlation between UKB fields and resting state FC based on the Power atlas, continued. [1]BMI, weight, and related fields were omitted as being redundant with waist circumference.

# UKB and HCP Intervention Fields

| Field | Mean | Std Dev | % Yes | % No | UKB Field ID |
|---|---|---|---|---|---|
| Non-modifiable/genetic | | | | | |
| Age (years) | 64.9 | 7.5 | | | 21003_2 |
| Sex (male) | | | 49.8 | 50.2 | 31_0 |
| Height (cm) | 170.1 | 8.7 | | | 50_0 |
| AD PRS[1] | 0.02 | 0.96 | | | 26206_0 |
| Modifiable risk factors | | | | | |
| Current Tobacco | | | 3.6 | 96.4 | 1239_0 |
| Past Tobacco | | | 76.1 | 23.9 | 1249_0 |
| Phys Activity (days) | 1.85 | 1.87 | | | 904_0 |
| Outdoor Summer (hrs) | 2.72 | 3.44 | | | 1050_0 |
| Outdoor Winter (hrs) | 1.42 | 1.53 | | | 1060_0 |
| Watching TV (hrs) | 1.46 | 3.68 | | | 1070_0 |
| Sleep Duration (hrs) | 7.15 | 0.98 | | | 1160_0 |
| Health Rating (1-4)[2] | 1.92 | 0.65 | | | 2178_0 |
| Physiological measurements | | | | | |
| Waist Circumference (cm) | 88.4 | 12.3 | | | 48_0 |
| Diabetes | | | 2.9 | 97.1 | 2443_0 |
| Pulse Rate (bpm) | 67.7 | 10.9 | | | 102_0 |
| Diastolic BP (mmHg) | 82.1 | 10.7 | | | 4079_0 |
| Systolic BP (mmHg) | 137.1 | 18.1 | | | 4080_0 |
| RBC Count ($10^{12}$/L) | 4.52 | 0.43 | | | 30010_0 |
| Hb Concentration (g/dL) | 14.2 | 1.25 | | | 30020_0 |
| Hand Grip Strength (kg) | 31.9 | 11.3 | | | 46_0 |

Table 5: Non-modifiable/genetic factors, modifiable lifestyle factors, and physiologic measurements from the UKB dataset included in the VAE model. [1]Polygenic risk score. [2]Lower health rating is better.

| Field | Mean | Std Dev | % Yes | % No |
|---|---|---|---|---|
| Non-modifiable/genetic | | | | |
| Age (years) | 28.5 | 3.69 | | |
| Sex (male) | | | 45.4 | 54.6 |
| Height (in) | 67.5 | 3.8 | | |
| Race (Caucasian) | | | 77.2 | 22.8 |
| Modifiable risk factors | | | | |
| Any Tobacco 7 Days | 5.6 | 18.8 | | |
| Alcoholic Drinks 7 Days | 4.6 | 6.4 | | |
| PSQI[1] | 4.5 | 2.7 | | |
| Physiological measurements | | | | |
| Weight (lbs) | 158 | 32 | | |
| BMI (kg/$m^2$) | 24.2 | 3.5 | | |
| Diastolic BP (mmHg) | 75.1 | 10.1 | | |
| Systolic BP (mmHg) | 121.4 | 12.6 | | |
| Hematocrit (%) | 43.2 | 4.9 | | |
| Hand Grip Strength (lbs) | 116 | 11 | | |

Table 6: Non-modifiable/genetic factors, modifiable lifestyle factors, and physiological measurements from the HCP dataset included in the VAE model. [1]Pittsburgh Sleep Quality Index; lower scores better.

## Top Positively and Negatively Weighted Connections for AD Risk

| Negatively-Weighted for AD Connections | | | | |
|---|---|---|---|---|
| ROI 1 | ROI 2 | Network 1 | Network 2 | Weight |
| 131 | 135 | VIS | VIS | -0.51 |
| 95 | 107 | DMN | DMN | -0.48 |
| 131 | 139 | VIS | VIS | -0.47 |
| 209 | 260 | SUB | UNK | -0.44 |
| 169 | 253 | FRNT | UNK | -0.44 |
| 236 | 246 | UNK | UNK | -0.44 |
| 209 | 221 | SUB | DRSL | -0.43 |
| 58 | 209 | AUD | SUB | -0.43 |
| 129 | 144 | VIS | VIS | -0.42 |
| 209 | 225 | SUB | DRSL | -0.42 |
| Positively-Weighted for AD Connections | | | | |
| ROI 1 | ROI 2 | Network 1 | Network 2 | Weight |
| 73 | 124 | DMN | MEM | 0.50 |
| 71 | 248 | DMN | UNK | 0.48 |
| 73 | 142 | DMN | VIS | 0.47 |
| 23 | 211 | SMTH | SUB | 0.46 |
| 178 | 247 | FRNT | UNK | 0.46 |
| 121 | 151 | MEM | VIS | 0.43 |
| 28 | 211 | SMTH | SUB | 0.43 |
| 32 | 211 | SMTM | SUB | 0.43 |
| 200 | 205 | SUB | SUB | 0.43 |
| 26 | 211 | SMTH | SUB | 0.42 |

Table 7: List of top ten connection weights positively and negatively correlated with AD in the aggregated ADNI AD risk model. ROIs are numbered starting from zero in the Power atlas. Note the large number of VIS network connections negatively correlated with AD risk. In contrast, there are many SUB and DMN connections with positive correlation with AD risk.

# Functional Networks in the Power Atlas

**Brain Functional Networks**

| Label | ROIs | | Label | ROIs | |
|---|---|---|---|---|---|
| 0 | 0-29 | Somatomotor Hand (SMTH) | 7 | 156-180 | Frontoparietal (FRNT) |
| 1 | 30-34 | Somatomotor Mouth (SMTM) | 8 | 181-198 | Salience (SAL) |
| 2 | 35-48 | Cinguloopercular (CNG) | 9 | 199-211 | Subcortical (SUB) |
| 3 | 49-61 | Auditory (AUD) | 10 | 212-220 | Ventral Attention (VTRL) |
| 4 | 62-119 | Default Mode (DMN) | 11 | 221-231 | Dorsal Attention (DRSL) |
| 5 | 120-124 | Memory (MEM) | 12 | 232-235 | Cerebellar (CB) |
| 6 | 125-155 | Visual (VIS) | 13 | 236-263 | Uncertain (UNK) |

Table 8: Regions, abbreviations, and functional networks in the Power atlas.

# Simulation Experiments

The ability of our counterfactual VAE model to accurately uncover the relationships between latent factors, an intermediate phenotype resembling FC, and outcome risk was tested in simulation experiments with a known ground truth. The simulation consisted of 6 latent factors, labeled $a$ through $f$, which affected both the intermediate phenotype $(\mathbf{x}, \mathbf{y})$ and outcome risk $y$. In order to match our real FC-based analysis, two synthetic datasets, $\mathcal{D}_X$ and $\mathcal{D}_Y$, with $\mathcal{D}_X$ consisting of 1000 samples and $\mathcal{D}_Y$ consisting of 100 samples, were created. Outcome risk $y$ was known only for the $\mathcal{D}_Y$ samples, however both $\mathbf{x}$ and $\mathbf{y}$ were the same functions of the latent factors. The synthetic data was instantiated as follows:

$$\begin{aligned} a, b, c, d, e, f &\sim \mathcal{N}(0, 1) \\ y &= a + b + a * b + c^2 - d \\ \mathbf{a}, \mathbf{b}, \mathbf{c}, \mathbf{d}, \mathbf{e}, \mathbf{f} &\sim \mathcal{N}(\mathbf{0}, \mathbf{I}) \\ \mathbf{x}, \mathbf{y} &= a\mathbf{a} + b\mathbf{b} + c\mathbf{c} + d\mathbf{d} + e\mathbf{e}, \end{aligned} \tag{2}$$

where $\mathbf{x}$ and $\mathbf{y}$ have 1000 features ($\mathbf{x}, \mathbf{y} \in \mathbb{R}^{1000}$) and are functions of $a, b, c, d, e$, while the outcome $y$ (a scalar) is a non-linear function of $a, b, c, d$. FC principal components $\mathbf{a}, \mathbf{b}, \mathbf{c}, \mathbf{d}, \mathbf{e}, \mathbf{f} \in \mathbb{R}^{1000}$ are sampled once, and re-used for every synthetic subject, while a new sample of $a, b, c, d, e, f$ is created for every subject. For the simulation experiments, we assume that $a, b, c, f$ are observed latent factors while $d, e$ are unobserved. Therefore both $\mathbf{x}, \mathbf{y}$ and $y$ are functions of both observed and unobserved latent factors.

Our goal in the simulation experiments was to determine the relationship between the observed latent factors $a, b, c, f$ and outcome risk $y$. We performed this in two ways. First, as a baseline, we regressed $\mathbf{y}$ on observed features $a, b, c, f$, to determine the approximate values of $a, b, c, f$ for each sample based on the relationship between $a, b, c, f$ and $\mathbf{a}, \mathbf{b}, \mathbf{c}, \mathbf{f}$ and $\mathbf{y}$. Then, assuming outcome risk $y$ was a second-order polynomial in $a, b, c, f$, we regressed the outcome risk $y$ on the coefficients determined in the previous regression, thereby estimating the functional relationship between $y$ and the latent factors $a, b, c, f$. Regression was performed using the Ridge model of scikit-learn package using three different values of the regularization parameter $\alpha$ (Pedregosa et al., 2011). The initial regression coefficients corresponding to $\mathbf{a}$ through $\mathbf{f}$ were estimated on the larger dataset $\mathcal{D}_X$, then evaluated for the dataset $\mathcal{D}_Y$ for which outcome risk $y$ was available.

After determining the coefficients using the baseline two-stage regression, we then attempted to determine the coefficients of outcome risk based on latent factors using the intervention VAE model. We began

by fitting an MLP on $\mathcal{D}_Y$ to predict $y$ from $\mathbf{y}$. The MLP was a simple two-layer function with a size 30 hidden layer. The MLP was trained for 2000 epochs using the Adam optimizer with a learning rate of $10^{-4}$ and L2 regularization of $10^{-5}$. Then, we trained the intervention VAE on the dataset $\mathcal{D}_X$ conditioned upon observed latent factors $a, b, c, f$. Once the VAE model was trained, we intervened on each of the latent factors in turn and determine the change in counterfactual outcome risk $\hat{y}$ compared to $y$ before intervention. We then fit coefficients to each of the latent factors, assuming the true function is a second-degree polynomial in the latent factors. The mean squared error of the coefficients determined using VAE was then compared to the coefficients found using the two-stage regression approach outlined above. The synthetic data was simulated ten times and coefficients were estimated for each simulation.

## Simulation Results

The results of our simulation experiments are shown in Figure 1. We find that due to the ability of the VAE to create a highly reliable model on the larger synthetic dataset $\mathcal{D}_X$, the ability to estimate coefficients for $y$ from $\mathcal{D}_Y$ was greatly enhanced. The VAE was able to correctly estimate those coefficients $(a, b, a{*}b, c^2)$ that were non-zero and set to zero those coefficients which were observed but not important for outcome risk $(f)$. On the other hand, the two-stage regression we were comparing against was highly dependent on the regularization hyperparameter used for the regression. Using a large regularization hyperparameter $\alpha = 100$, the two-stage regression was able to set $f$ to be correctly equal to zero, but the other coefficients were found to be too small, around or less than 0.5 instead of the ground truth of one. Decreasing the regularization parameter allowed the coefficients of $a, b, a * b, c^2$ to reach their correct values, but with the downside that $f$ was no longer exactly zero but had a distribution of values around zero. In any case, we found the variance of the coefficients estimated using two-stage regression to be much larger than for the interventional VAE model. Mean squared error for the coefficients for the VAE model and each two-stage regression are given in Table 9.

| Coefficient MAE (True Value) | | | | | |
|---|---|---|---|---|---|
| | $a$ (1) | $b$ (1) | $a * b$ (1) | $c^2$ (1) | $f$ (0) |
| Reg. $\alpha = 1$ | $.106 \pm .092$ | $.052 \pm .060$ | $.095 \pm .085$ | $.019 \pm .020$ | $.906 \pm .472$ |
| Reg. $\alpha = 10$ | $.017 \pm .022$ | $.017 \pm .025$ | $.032 \pm .025$ | $.005 \pm .006$ | $.092 \pm .084$ |
| Reg. $\alpha = 100$ | $.314 \pm .079$ | $.247 \pm .065$ | $.332 \pm .138$ | $.101 \pm .051$ | $\mathbf{.001 \pm .002}$ |
| Inter. VAE | $\mathbf{.001 \pm .002}$ | $\mathbf{.001 \pm .002}$ | $\mathbf{.004 \pm .002}$ | $\mathbf{.001 \pm .001}$ | $\mathbf{.002 \pm .004}$ |

Table 9: Results of simulation experiments. Mean squared error for coefficients of latent factors leading to outcome risk determined using the intervention VAE or two-stage regression model. Three regression models are shown, using three different settings of the L2 regularization hyperparameter $\alpha$.

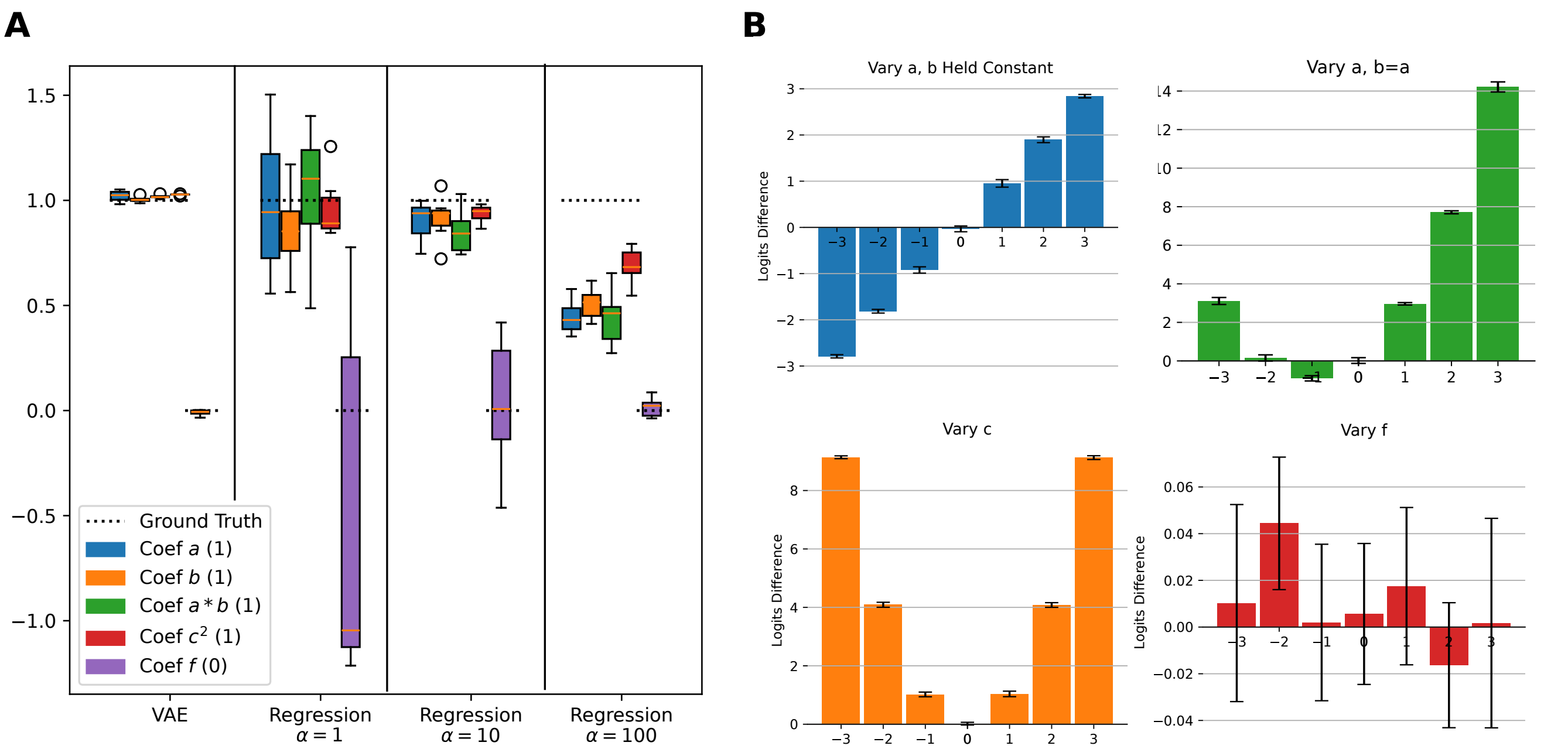


Figure 1: Results of the simulation experiments comparing use of the intervention VAE to two-stage regression in order to find the coefficients of a latent factor model of outcome risk. Part (A) shows estimates of coefficients for $a, b, a*b, c^2, f$ using either VAE or two-stage regression at three different settings for the regularization hyperparameter $\alpha$. It can be seen that the VAE is able to estimate coefficient values almost exactly while the two-stage regression has more variance and is highly dependent on the value of the regularization hyperparameter. (B) shows the method of estimating coefficients in the VAE model by intervening on each coefficient value and observing the resulting change in outcome risk.

| Group | # Subjects | CDR | MMSE | Age |
|---|---|---|---|---|
| CN Females | 347 | $0.01 \pm 0.04$ | $29.83 \pm 0.48$ | $71.01 \pm 5.96$ |
| CN Males | 235 | $0 \pm 0$ | $29.72 \pm 0.60$ | $72.05 \pm 6.21$ |
| AD Females | 68 | $9.26 \pm 3.99$ | $16.44 \pm 6.55$ | $73.41 \pm 8.49$ |
| AD Males | 87 | $7.87 \pm 3.86$ | $19.66 \pm 4.98$ | $74.73 \pm 5.82$ |

Table 10: Mean and standard deviations for clinical dementia rating (CDR), mini mental state examination (MMSE), and ages for the four study groups (CN females, CN males, AD females, and AD males) comprising our ADNI cohort. Each bootstrap repetition sampled 68 subjects from each group to create a balanced sample. A single scan was used from each subject. Cognitive test information and ages are those acquired at the time the scan was taken.

## ADNI Cohort Ages and Cognitive Assessment Scores

To better quantify amount or lack of AD burden in our ADNI cohort, we provide cognitive test information and ages for the 272 subjects whose scans make up our ADNI cohort in Table 10. Clinical dementia rating (CDR), mini mental state examination (MMSE), and ages are provided. The CDR is a widely used, standardized instrument for assessing the severity and progression of dementia (Morris, 1993). The CDR evaluates six cognitive and functional domains that are typically affected by dementia: memory, orientation, judgment/problem solving, community affairs, home and hobbies, and personal care. Each domain is rated on a five point scale, with zero for no impairment and three for severe dementia. The MMSE is a brief, standardized cognitive screening tool used to assess global cognitive function and detect cognitive impairment associated with conditions such as Alzheimer's disease and other dementias (Folstein et al., 2014). The MMSE consists of 11 items assessing several key cognitive domains, with a maximum total score of 30 points. A higher score indicates less impairment; a greater score indicates more severe dementia.

## Impact of Bootstrap Repetition Number on AD Risk Model

To assess the reproducibility of the ADNI Alzheimer's disease (AD) risk model, we compared the model weights obtained using 20 bootstrap iterations with those derived from a larger bootstrap sample of 100 iterations. The results are presented in Figure 2. Overall, the weight estimates from the smaller bootstrap sample were highly consistent with those from the larger sample, with discrepancies below $5\%$ for the majority of functional network connections. The largest deviation was observed for the MEM–MEM connection, which exhibited a relative difference of $+14.5\%$.

In each bootstrap repetition, we con-struct a class-balanced training set of 272 subjects by sampling 68 individuals with replacement from each of four strata, namely CN males, CN females, AD males, and AD

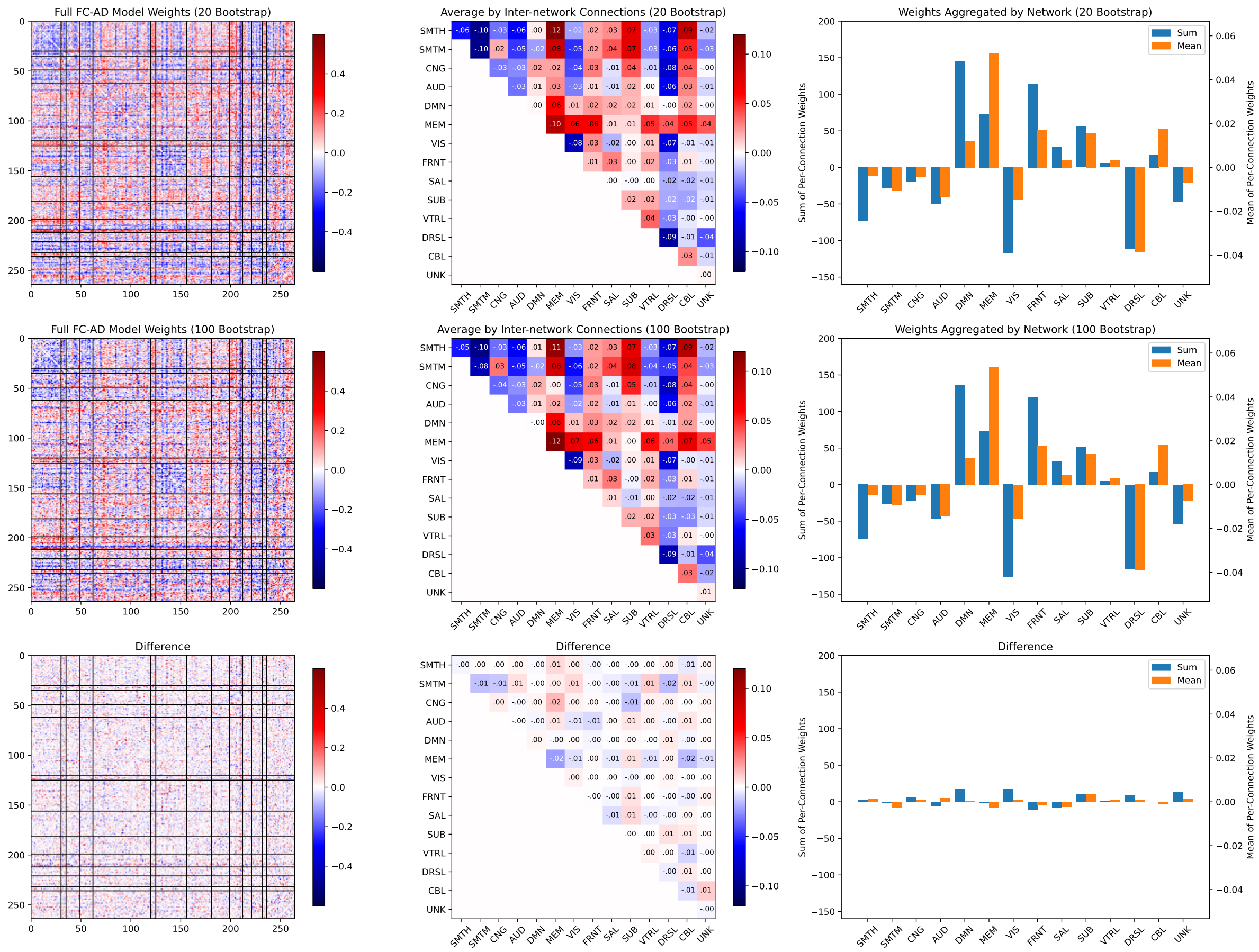


Figure 2: Effect of bootstrap sample size on functional connectivity–associated model weights for AD prediction in the ADNI dataset. The top row presents model weights estimated using 20 bootstrap repetitions, and the middle row shows the corresponding weights obtained from 100 repetitions. The bottom row depicts the difference between the two sets of estimates. Overall, the spatial patterns of weights are qualitatively consistent across bootstrap sample sizes, indicating that reducing the number of repetitions to 20 does not substantially compromise the stability of the estimated model weights.

females. The full ADNI cohort available to us comprised 87 AD males, 68 AD females, 235 CN males, and 347 CN females. Because AD females are the smallest stratum, all AD female subjects are necessarily included in every repetition, whereas the other strata vary across bootstraps due to resampling. Our stratified bootstrap is designed to control for diagnosis and sex imbalance, so the FC weight estimates are not dominated by the much larger CN strata. Although each repetition includes 272 subjects, three of the four strata are resampled differently across repetitions, so the aggregate estimates reflect broad coverage of the cohort rather than a single fixed subsample.

Although our FC-based AD risk model for the ADNI dataset is derived using ensemble averaging, it is nonetheless informative to characterize the variability of the model parameters across bootstrap samples. Figure 3 summarizes this variability by reporting the dispersion of the learned weights at both the

individual FC edge level and when aggregated within canonical functional networks. We acknowledge the need to report the full distribution of bootstrap-derived weights. In our pipeline, we require a single set of AD-risk weights because the downstream interventional VAE generates a large number of synthetic FC samples, and each sample must be mapped to one AD-risk logit in a consistent and scalable manner. For this reason, we use aggregation to obtain one stable predictive model, and weights that are not consistently informative across bootstrap repetitions tend to shrink toward zero in the aggregate.

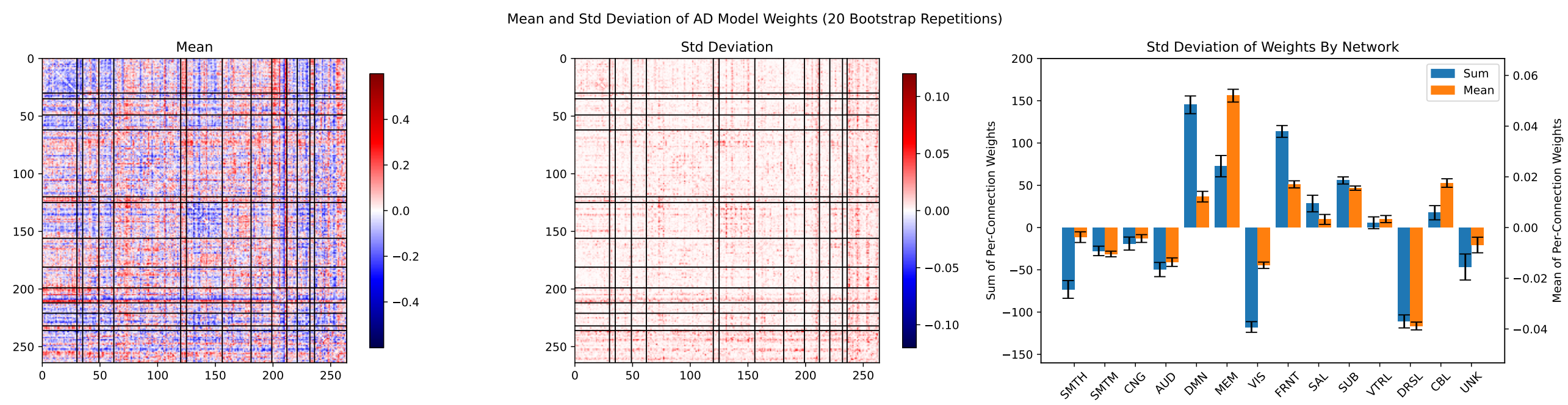


Figure 3: Mean and standard deviations of weights for AD risk across subject FCs for 20 bootstrap repetitions in the ADNI dataset.

# Intervention on Factors That Do Not Affect FC

Given that our framework enables targeted interventions on physiological and modifiable lifestyle factors that influence FC – and subsequently quantifies their impact on FC-mediated AD risk – it is important to assess model behavior when intervening on variables that do not affect FC. In Supplementary Section Simulation Experiments, we evaluate this scenario using a synthetic factor $f$ that is present in the dataset but is constructed to influence neither FC nor the continuous ground-truth AD label. As shown in Supplementary Figure 1, interventions on factors $a$, $b$ and $c$ – which do affect both FC and the AD label – are accurately recovered by the model; in contrast, intervention on the null factor $f$ produces no meaningful change in the predicted AD-risk logits, consistent with expectations.

It is also informative to examine this behavior in real data, where multiple variables exhibit minimal correlation with FC (Supplementary Tables 1–4). To evaluate the model's capacity to appropriately discount interventions on such uninformative factors, we selected four variables with near-zero empirical correlations: Number of Operations ($\rho = 0.04$), Fresh Fruit Intake ($\rho = 0.03$), Oily Fish Intake ($\rho = 0.02$), and Tense/High-Strung Feelings ($\rho = 0.02$). Three of these variables are continuous, whereas Tense/High-Strung Feelings is a binary categorical field. The intervention results, presented

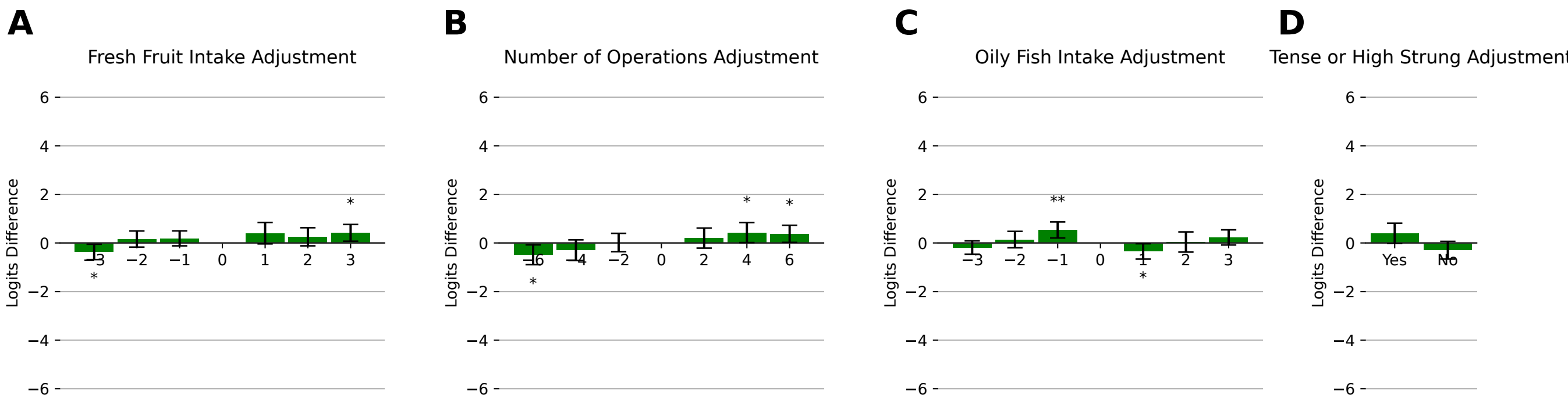


Figure 4: Intervention on modifiable lifestyle factors that have minimal correlation with FC. These factors produced comparatively small changes in the FC-mediated AD-status score. Correlations with FC can be found in Supplementary Tables 1-4.

in Supplementary Figure 4, show that manipulating these low-correlation factors yields no appreciable changes in predicted AD risk.

These findings demonstrate that the model appropriately constrains causal attributions, responding robustly to factors that meaningfully influence FC while remaining insensitive to those with negligible FC associations.

# Additional Supplementary Tables

| Group Difference | Real Mean | Synthetic Mean | MSE (Real/Synthetic) |
|---|---|---|---|
| Age (UKB) | $0.0592 \pm 0.0114$ | $0.0437 \pm 0.0151$ | $0.0117 \pm 0.0014$ |
| Age (HCP) | $-0.0124 \pm 0.0210$ | $-0.0178 \pm 0.0113$ | $0.0074 \pm 0.0056$ |
| Sex (UKB) | $0.0684 \pm 0.0098$ | $0.0479 \pm 0.0064$ | $0.0138 \pm 0.0049$ |
| Sex (HCP) | $0.0048 \pm 0.0084$ | $0.0099 \pm 0.0074$ | $0.0102 \pm 0.0043$ |
| BMI (UKB) | $-0.0433 \pm 0.0132$ | $-0.068 \pm 0.0103$ | $0.0125 \pm 0.0068$ |
| BMI (HCP) | $-0.0008 \pm 0.0118$ | $0.0064 \pm 0.0097$ | $0.0109 \pm 0.0078$ |

Table 11: Mean squared errors quantifying discrepancies between empirical and VAE-reconstructed group-difference FC matrices for age, sex, and BMI in the HCP and UKB cohorts (see Figure 6).

# Ablation Experiments

Ablation experiments were performed to test some of the individual components (as represented by loss terms) in the InterventionVAE model. For this purpose, synthetic data was generated with a known ground truth that comprised of a response feature, a covariate feature, and input features. A set of 2000

synthetic subjects was generated, split into 1000 training set subjects and a 1000 subject hold-out test set. The response feature was picked from a standard normal distribution, as were the covariate and input features. The covariate feature was made to be correlated with the response feature at a level of $\rho = 0.8, 0.6, 0.4$ through the formula $x_{cov} = \rho x_{res} + \sqrt{1-\rho^2} x_{unc}$, where $x_{unc}$ was the uncorrelated component also taken from a standard normal distribution. The input features were then made to be correlated with the covariant features in a similar manner, at a level $\rho$ equal to the correlation between the response and covariate features. A linear model was then constructed to predict the response feature from the input feature, similar to how AD risk was modeled to be predicted by FC in the main part of the manuscript.

An InterventionVAE model was now constructed to synthesize input features from covariate features. This is analogous to how in the main part of the manuscript synthetic FC was generated based on intervention to demographic or questionnaire field values. The parameters of the InterventionVAE model were set to the values used for intervention on risk factors in the main part of the paper, selected via random grid search for those values that best recapitulated FC differences between different risk factors on real data. These values are given in Supplementary Table 12.

| | |
|---|---|
| Logistic Regression (discrete) C-value | 100 |
| Ridge Regression (continuous) alpha | 100 |
| Batch Size | 1000 |
| Latent Dimension | 100 |
| Learning Rate | $5 \cdot 10^{-5}$ |
| Number of Epochs | 10000 |
| Weight Decay | 0 |
| Covariance Loss Multiplier | 10000 |
| Mean Loss Multiplier | 1 |
| Reconstruction Loss Multiplier | 1000 |
| Decorrelation Loss Multiplier | 10 |
| Prediction Loss Multiplier | 100 |

Table 12: Default parameter values for InterventionVAE model.

In the ablation experiments, it was chosen to look at decorrelation loss and prediction loss in particular, and how they affect the prediction of the response variable from intervention on covariates via the input variable. In the main paper, this is analogous to how risk factors affect AD risk through their effect on FC. The values of decorrelation loss multiplier and prediction loss multiplier were varied between $0, 0.001, 0.01, 0.1, 1, 10, 100, 100$. An InterventionVAE model was trained for each value of the parameter, then the hold out test set of covariates was used to generate synthetic input variables. The input feature to response feature model was then used to estimate the value of the response features, and the RMSE error was recorded. Experiments were repeated 100 times and 95% confidence bands were calculated.

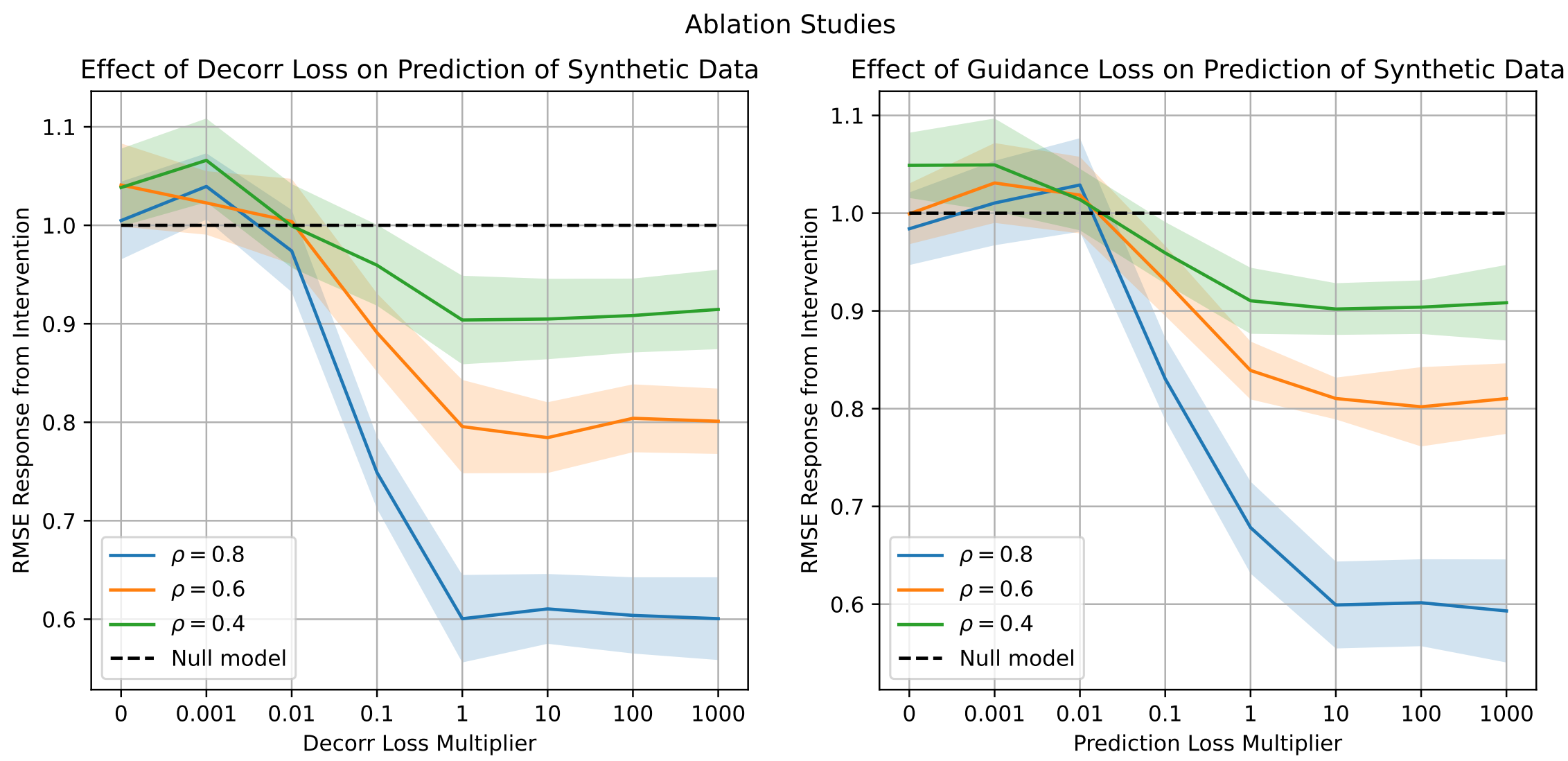


Figure 5: Results of ablation experiments. It is seen that complete ablation of either the decorrelation loss component or the predictive component of the InterventionVAE results in loss of accurate ability to predict input features from covariates.

The results are shown in Supplementary Figure 5. We see that the results for complete ablation of either the decorrelation loss or prediction loss results in prediction no better than the null model. At a moderate level of multiplier, however, the InterventionVAE recovers the relationship between covariates and input features, thus allowing prediction of response feature based on synthetic input features derived from simulating covariate effects on input features. We conclude that both decorrelation loss and prediction loss are needed for accurate simulation of input features (FC in the main part of the manuscript) from covariates (demographics or risk factors in the main part of the manuscript).

# References

Folstein, M. F., Folstein, S. E., & McHugh, P. R. (2014, February). Mini-Mental state examination [Title of the publication associated with this dataset: PsycTESTS Dataset].

Morris, J. C. (1993). The clinical dementia rating (CDR): Current version and scoring rules. *Neurology*, *43*(11), 2412–2414.

Pedregosa, F., Varoquaux, G., Gramfort, A., Michel, V., Thirion, B., Grisel, O., Blondel, M., Prettenhofer, P., Weiss, R., Dubourg, V., Vanderplas, J., Passos, A., Cournapeau, D., Brucher, M., Perrot, M., & Duchesnay, E. (2011). Scikit-learn: Machine learning in Python. *Journal of Machine Learning Research*, *12*, 2825–2830.
